# Synthesis, Antitumor and Antiviral *In Vitro* Activities of New Benzotriazole-Dicarboxamide Derivatives

**DOI:** 10.3389/fchem.2021.660424

**Published:** 2021-05-04

**Authors:** Roberta Ibba, Sandra Piras, Paola Corona, Federico Riu, Roberta Loddo, Ilenia Delogu, Gabriella Collu, Giuseppina Sanna, Paola Caria, Tinuccia Dettori, Antonio Carta

**Affiliations:** ^1^ Department of Chemistry and Pharmacy, University of Sassari, Sassari, Italy; ^2^ Department of Biomedical Sciences, Section of Microbiology and Virology, University of Cagliari, Cittadella Universitaria, Cagliari, Italy; ^3^ Department of Biomedical Sciences, Section of Biochemistry, Biology and Genetics, University of Cagliari, Cittadella Universitaria, Cagliari, Italy

**Keywords:** antimetabolites, antiproliferative activity, antivirals, benzotriazole derivatives, dicarboxamides, coxsackievirus

## Abstract

Cancer and viral infections continue to threaten humankind causing death worldwide. Hence, the discovery of new anticancer and antiviral agents still represents a major scientific goal. Heterocycles designed to mimic the chemical structure of natural pyrimidines and purines have been designed over the years, exerting their activity acting as false substrates on several different targets. We reported a series of bis-benzotriazole-dicarboxamide derivatives which inhibit viral helicase of poliovirus, and hence we planned structure modifications to obtain different series of new dicarboxamides. Here, the synthesis and characterization of 56 new compounds: 31 bis-benzotriazole dicarboxamides and 25 mono-substituted acidic derivatives are reported. The synthesized compounds were tested for their antiviral and antitumor activity. Mostly, compounds **4a**, **4c** and **4d** showed antiviral activity against tested Picornaviruses, Coxsackievirus B5 and Poliovirus-1. Likewise, four derivatives (**3b**, **3d**, **4d**, **9b**) showed notable antiproliferative activity inhibiting cell growth in two distinct antitumor screenings. Compound **3b** was selected as the antitumor lead compound for the wide range of activity and the potency proved. The lead compound was proved to induce apoptosis in SK-MES1 tumor cells, in a dose-dependent manner.

## Introduction

**FIGURE 1:**
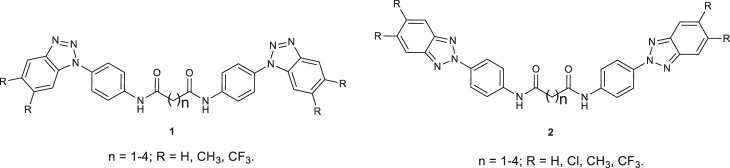
Previously reported *N*,*N′*-bis-[4-(1*H*(2*H*)-benzo [*d*][1,2,3]triazol-1(2)-yl)phenyl]alkyl dicarboxamides **1** and **2**.

Among the various diseases that affect humankind, cancer and viral infections remain great threats to human health worldwide. Therefore, the identification of novel anticancer and antiviral agents remains one of the most pressing health problems and the most stimulating cue for research. Polycyclic benzo-fused azoles can mimic the chemical structure of natural pyrimidines and purines, exerting their biological activity acting as false substrates, resulting in antitumor, antibacterial or antiviral agents. Considering the similar mechanism of action of the latter, cross-activity has been highlighted for several molecules (Kaufman, 1980; Matsuda et al., 1999; Ramachandran et al., 2011; Brüning et al., 2012). Therefore, anticancer drugs have been proved active against viruses (Kaufman, 1980) and antiviral agents have shown antimetabolite activity (Bergman et al., 2002; De Clercq, 2004; Brüning et al., 2012). 5-fluorouracil is a well-known example; it is a widely used anticancer agent which acts as antimetabolite. It was proved active against several viruses such as foot and mouth disease virus (Pariente et al., 2003; Agudo et al., 2009), herpes simplex virus (Dragún et al., 1990), poliovirus (Cooper, 1964). Purine-like antimetabolites enter the metabolic pathway acting as false substrates or mimicking the natural building blocks of RNA and DNA (De Clercq, 2013). For these features, besides human-cell targets, viral enzymes involved in RNA and DNA synthesis, such as RNA-dependent-RNA-polymerases (RdRp) (Furuta et al., 2017), helicases (Carta et al., 2007), proteases (Kneller et al., 2019), may be targeted by this class of derivatives. Benzotriazole (BT) derivatives have been widely studied for their broad biological activity (Briguglio et al., 2015). BT derivatives may mimic purine ring acting as antimetabolites interfering with purine metabolism but may also bind the enzymes that naturally interact with purines, mimicking the natural substrate, resulting in potential antitumor (Al-Soud et al., 2003), antibacterial (Sanna et al., 1992; Carta et al., 2011; Ochal et al., 2013), antifungal (Patel et al., 2010) or antiviral (Borowski et al., 2003) agents. In the process of new drug discovery, we designed several promising antiviral active derivatives bearing the benzotriazole scaffold (Ibba et al., 2018; Piras et al., 2019a; Piras et al., 2019b; Sanna et al., 2020). Amongst them, benzotriazole derivatives depicted in Figure 1 have been designed, synthesized and proved as antiviral agents against two tested Picornaviruses (Coxsackievirus B2 and Poliovirus Sb-1) (Carta et al., 2007). Bis-benzotriazole-dicarboxamide derivatives (Figure 1) were proved to exert their antiviral activity as false substrates binding the Poliovirus helicase in *in silico* assays, performed on the 3D model of the target protein (Carta et al., 2007). Bis-benzotriazole-dicarboxamide derivatives from series **2** turned out as the most active with EC_50_ values ranging from 4 to 33 μM against Coxsackievirus B2.

**FIGURE 2:**
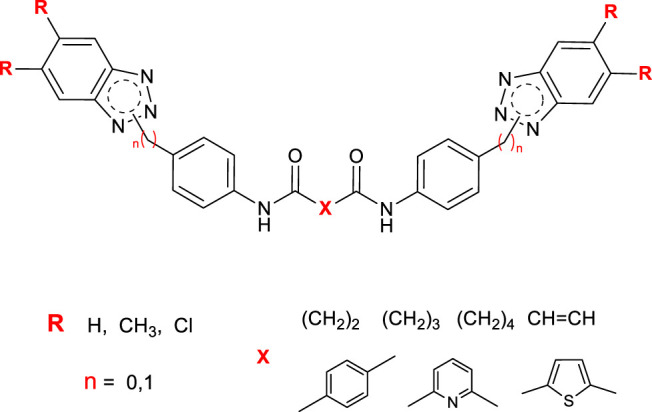
General structure of newly designed derivatives, applied structure-modifications are outlined in red.

Based on this, we designed and synthesized a new series of bis-benzotriazole-dicarboxamide derivatives as potential anti-Picornavirus agents, acting as false substrates. The structural modifications applied to the main scaffold are shown in red in Figure 2. We evaluated the substitution on the benzotriazole scaffold in positions 5 and 6 (**R**). We also considered the length and the role of the linker amidst the two amidic groups (**X**), including saturated or unsaturated aliphatic or small aromatic linkers. The insertion of a CH_2_ bridge between the BT scaffold and the aromatic moiety directly connected to the BT nitrogen was also attempted.

All the synthesized compounds were tested against a panel of Picornaviruses and several cell lines to assess the cytotoxicity. Only a few of the designed molecules turned out active against tested viruses, while some were selected for antiproliferative assays, based on the known antiviral-anticancer correlations. Notably, four derivatives (**3b**, **3d**, **4d**, **9b**) showed interesting antiproliferative activity inhibiting cell growth and inducing apoptosis. Compound **3b** was selected as the lead compound to be subjected to further structure-modifications in order to improve the antiproliferative activity.

## Materials and Methods

### Chemistry

Reaction progression, retention factors (R_*f*_) and purity of compounds were monitored by TLC using Merck F-254 commercial plates and a proper mixture of solvents as eluent. Melting points were taken in open capillaries in a Köfler hot stage or Digital Electrothermal melting point apparatus. ^1^H-NMR spectra were recorded on a Nuclear magnetic resonance (^1^H-NMR) spectra were determined in DMSO-*d*_6_ and were recorded with a Bruker Avance III 400 NanoBay (400 MHz) and a Varian XL-200 (200 MHz) instruments. Chemical shifts (*δ* scale) are reported in parts per million (ppm) downfield from tetramethylsilane (TMS) used as internal standard. The chemical shift values are reported in ppm (*δ*) and coupling constants (*J*) in Hertz (Hz). Signal multiplicities are represented as: s (singlet), d (doublet), t (triplet), and m (multiplet). The assignment of exchangeable protons (OH and NH) was confirmed by the addition of D_2_O. ^13^C-NMR were determined in DMSO-*d*_6_ and were recorded at 100 MHz with Bruker Advance III 400 NanoBay. Mass spectra were performed on combined Liquid Chromatograph-Agilent 1100 series Mass Selective Detector (MSD). Column chromatography was performed using 70–230 mesh (Merck silica gel 60). Petroleum ether (PE) refers to the fraction with b.p. 40–60°C. Elemental analysis was performed on a Perkin-Elmer 2400 instrument at “Laboratorio di Microanalisi”, Department of Chemistry and Pharmacy, University of Sassari, Italy, and the results were within ±0.4% of theoretical values.

### Biology

#### Cells and Viruses

Cell lines were purchased from American Type Culture Collection (ATCC). Cell lines supporting the multiplication of CV-B5 and Sb-1 are Monkey kidney (Vero-76) [ATCC CRL 1587 *Cercopithecus Aethiops*]. Viruses were purchased from the American Type Culture Collection (ATCC). *Picornaviridae*: Coxsackievirus [coxsackie type B5 (CV-B5), strain Faulkner (ATCC VR-185)], and human enterovirus C [poliovirus type-1 (Sb-1), Sabin strain Chat (ATCC VR-1562)]. Cell cultures were checked periodically for the absence of mycoplasma contamination with MycoTect Kit (Gibco). Viruses were maintained in our laboratory and propagated in appropriate cell lines. The viruses were stored in small aliquots at −80 °C until use.

#### Cytotoxicity Assays

Vero-76 cells were seeded in 96-well plates at an initial density of 5 × 10^5^ cells/ml, in Dulbecco’s Modified Eagle Medium (D-MEM) with L-glutamine and 25 mg/L kanamycin, supplemented with 10% FBS. Cell cultures were then incubated at 37°C in a humidified and 5% CO_2_ atmosphere, in the absence or presence of serial dilutions of test compounds. The test medium used for the cytotoxic assay as well as for antiviral assay contained 1% of the appropriate serum. Cell viability was determined after 48–96 h at 37°C by MTT method for Vero-76 (Pauwels et al., 1988).

#### Antiviral Assays

Compounds activity against CV-B5 and Sb-1 was determined by plaque reduction assays in infected cell monolayers. Briefly, monolayer of Vero-76 cells was grown overnight on 24-well plate. The cells were then infected for 2 h with 250 μl of proper virus dilutions to give 50–100 PFU/well. Following removal of unadsorbed virus, 500 μl of medium (D-MEM with L-glutamine and 4,500 mg/L D-glucose, supplemented with 1% inactivated FBS) containing 0.75% methyl-cellulose, with serial dilutions of test compounds, were added. The overlayed medium was also added to not treated wells as non-infection controls. Cultures were incubated at 37°C for 2 days for Sb-1 and 3 days for CV-B5 and then fixed with PBS containing 50% ethanol and 0.8% crystal violet, washed and air-dried. Plaques in the control (no inhibitor) and experimental wells were then counted (Sanna et al., 2015).

#### Antiproliferative Assays

Cell lines were purchased from American Type Culture Collection (ATCC). The absence of mycoplasma contamination was checked periodically by the Hoechst staining method. Exponentially growing leukemia and lymphoma cells were seeded at 1 × 10^5^ cells/ml in 96 well plates in RPMI-1640 medium, supplemented with 10% fetal bovine serum, 100 units/ml penicillin G, and 100 μg/ml streptomycin, and incubated at 37°C in a humidified, 5% CO_2_ atmosphere in the absence or presence of serial dilutions of test compounds. Cell viability was determined after 96 h at 37°C by the 3-(4,5-dimethylthiazol-2-yl)-2,5-diphenyl-tetrazolium bromide (MTT) method. Activity against solid-tumor derived cells was evaluated in exponentially growing cultures seeded at 5 × 10^4^ cells/ml and allowed to adhere for 16 h to culture plates before the addition of the drugs. Cell viability was determined by the MTT method 4 days later. Vincristine and doxorubicin were used as reference drugs.

#### Linear Regression Analysis

The extent of cell growth/viability and viral multiplication, at each drug concentration tested, were expressed as percentage of untreated controls. Concentrations resulting in 50% inhibition (CC_50_ or EC_50_) were determined by linear regression analysis.

#### NCI60 Human Tumor Cell Lines Screen

The *in vitro* anti-cancer screening was performed through the NCI60 Human Tumor Cell Lines Screen, provided by the Developmental Therapeutics Program of the National Cancer Institute (NCI, Bethesda, United States). A single dose of 10 µM of each compound is tested in the whole NCI60 cell panel. More details about the NCI60 screen methods are reported on the NCI website (https://dtp.cancer.gov ).

#### Apoptosis Assay

To assess which death mechanisms our compounds induced, cell apoptosis kit Annexin V/Propidium iodide (PI) double staining uptake (Invitrogen, Life Technologies, Italy) was used. **3b** compound was selected as lead and employed in our assay. Human lung cancer cells (SK-MES 1) were seeded at the density of 8 × 10^5^ cells/ml in 6-well plates (Corning, United States) with a complete medium (described in cell culture section). After overnight incubations, the cells were treated with or without different concentrations of **3b** for 96 h. Cells were then labeled with Annexin V and PI as previously described (Ibba et al., 2021). Stained cells were then analyzed by flow cytometry, measuring the fluorescence emission at 530 and 620 nm using 488 nm excitation laser (MoFloAstrios EQ, Beckman Coulter). Cell apoptosis was analyzed using Software Summit Version 6.3.1.1, Beckman Coulter.

### Experimental

#### Intermediates

5,6-Dichloro-1*H*-benzo [*d*][1,2,3]triazole (**21)** was synthesized as previously described (Carta et al., 2007), while 1*H*-Benzo [*d*][1,2,3]triazole **19**, 5,6-Dimethyl-1*H*-benzo [*d*][1,2,3]triazole **20**, 1- (Chloromethyl)-4-nitrobenzene and the diacyl dichlorides (**14a-g**) were commercially available. The intermediate 1 (2)-(4-Aminophenyl)-5,6-dichlorobenzo [*d*][1,2,3]triazoles (**13** and **15**) were prepared according to the procedures previously described (Carta et al., 2007).

#### General Procedure to Obtain (5,6-*R*)-1-(4-Nitrobenzyl)-1*H*-benzo[*d*][1,2,3]triazole (22–24)

To a mixture of proper benzotriazole (**19**) or 5,6-Dimethylbenzotriazole (**20**) or 5,6-Dichlorobenzotriazole (**21**) (2.7 mmol) and Cs_2_CO_3_ (2.7 mmol) in 30 ml of *N,N*-Dimethylformamide (DMF) a solution of 4-Nitrobenzylchloride (5.4 mmol) in 10 ml of DMF was added. The mixture was heated to 60°C for 48 h. After cooling down, the solution was filtered off *in vacuo* to remove the Cs_2_CO_3_ and the mothers were diluted with water until complete precipitation of the products. The filtered solid was in all cases a 3:1 mixture of two isomers: 5,6-*R*-1-(4-Nitrobenzyl)-1*H*-benzo [*d*][1,2,3]triazole and 5,6-*R*-2-(4-Nitrobenzyl)-2*H*-benzo [*d*][1,2,3]triazoles. The desired compounds 1-(4-Nitrobenzyl)-1*H*-benzo [*d*][1,2,3]triazole (**22**), 5,6-Dimethyl-1-(4-nitrobenzyl)-1*H*-benzo [*d*][1,2,3]triazole (**23**) and 5,6-Dichloro-1-(4-nitrobenzyl)-1*H*-benzo [*d*][1,2,3]triazole (**24**) were separated and purified by flash chromatography using a mixture of petroleum ether and ethyl acetate in a 8/2 (for **22** and **23**) or 7/3 (for **24**) ratio.

### 1-(4-Nitrobenzyl)-1*H*-Benzo[*d*][1,2,3]Triazole (22)

Compound **22** was obtained in 44% total yield; m.p. 126–128°C; TLC (petroleum ether/ethyl acetate 8/2); R_*f*_ = 0.13, ^1^H-NMR (200 MHz, DMSO-*d*_6_) *δ*: 8.22 (2H, d, *J* = 8.4, H-3′, 5′), 8.09 (1H, d, *J* = 8.2, H-4), 7.87 (1H, d, *J* = 8.2, H-7), 7.70–7.60 (1H, m, H_arom_), 7.54 (2H, d, *J* = 8.4, H-2′,6′), 7.47–7.43 (1H, m, H_arom_), 6.19 (2H, s, CH_2_). ^13^C-NMR (DMSO-*d*_6_) *δ*: 145.6 (C), 144.9 (C), 142.3 (C), 132.9 (C), 128.9 (2CH), 126.3 (2CH), 123.8 (2CH), 118.5 (CH), 110.5 (CH), 52.2 (CH_2_).

### 5,6-Dimethyl-1-(4-nitrobenzyl)-1*H*-Benzo[*d*][1,2,3]Triazole (23)

Compound **23** was obtained in 45% total yield; m.p.143–145°C; TLC (petroleum ether/ethyl acetate 8/2); R_*f*_ = 0.17; ^1^H-NMR (200 MHz, DMSO-*d*_6_) *δ*: 8.28 (2H, d, *J* = 8.8, H-3′,5′), 7.82 (1H, s, H-4) 7.62 (1H, s, H-7), 7.48 (2H, d, *J* = 8.8, H-2′,6′), 6.10 (2H, s, CH_2_), 2.35 (6H, s, 2CH_3_); ^13^C-NMR (DMSO-*d*_6_) *δ*: 145.6 (C), 144.9 (C), 142.2 (C), 132.9 (C), 131.9 (2C), 127.9 (2CH), 118.5 (2CH), 116.5 (2CH), 52.1 (CH_2_), 18.8 (2CH_3_).

### 5,6-Dichloro-1-(4-Nitrobenzyl)-1*H*-Benzo[*d*][1,2,3]Triazole (24)

Compound **24** was obtained in 32% total yield; m.p.158–160°C; TLC (petroleum ether/ethyl acetate 7.5/2.5); R_*f*_ = 0.42; ^1^H-NMR (400 MHz, CDCl_3_) *δ*: 8.21 (3H, d, *J* = 7.8, H-3′,5′, H-4), 7.88 (1H, s, H-7), 7.51 (2H, d, *J* = 8.6, H-2′,6′), 6.03 (2H, s, CH_2_). ^13^C-NMR (DMSO-*d*_6_) *δ*: 145.9 (C), 145.3 (C), 142.5 (C), 133.8 (2C), 132.6 (C), 128.6 (2CH), 126.5 (2CH), 124.3 (2CH), 52.0 (CH_2_).

#### General Procedure to Obtain 4-((5,6-*R*-1*H*-Benzo[*d*][1,2,3]Triazol-1-yl)Methyl)aniline (16–18)

To a mixture of proper compound **22** or **23** (1.77 mmol) in Ethanol (20 ml), and Hydrated hydrazine in ratio 1:20 (35.4 mmol), Pd/C in ratio 1:0.1 w/w was added. The mixture was stirred and heated at 80°C for 1 h. Palladium on carbon was filtered off when the solution was still hot, and the mothers obtained were concentrated into half volume. By cooling down, the resulting solid was filtered out *in vacuo*. The solid compound was washed twice with diethyl ether (20 ml) and subsequently crystallized from Ethanol. Derivative **24** (1.77 mmol) was solved in Ethanol (100 ml) and reduced with Methylhydrazine in 1/10 molar ratio, at 100°C in autoclave for 48 h.

### 4-((1*H*-Benzo[*d*][1,2,3]Triazol-1-yl)methyl)aniline (16)

Compound **16** was obtained in 78% total yield; m.p. 151–153°C; TLC (petroleum ether/ethyl acetate 4/6); R_*f*_ = 0.47; ^1^H-NMR (200 MHz, DMSO-*d*_6_) *δ*: 8.03 (1H, d, *J* = 8.4, H-4), 7.81 (1H, d, *J* = 8.4, H-7), 7.50 (1H, t, H-5), 7.41 (1H, t, H-6) 7.07 (2H, d, *J* = 8.2, H-3′,5′), 6.49 (2H, d, *J* = 8.2, H-2′,6′), 5.74 (2H, s, CH_2_), 5.15 (2H, s, NH_2_). ^13^C-NMR (DMSO-*d*_6_) *δ*: 149.2 (C), 145.5 (C), 132.4 (C), 132.8 (C), 128.7 (2CH), 126.6 (2CH), 119.7 (CH), 114.6 (2CH), 110.3 (CH), 52.3 (CH_2_).

### 4-((5,6-Dimethyl-1*H*-Benzo[*d*][1,2,3]Triazol-1-yl)Methyl)aniline (17)

Compound **17** was obtained in 88% total yield; m.p. 184–186°C; TLC (petroleum ether/ethyl acetate 6/4); R_*f*_ = 0.17; ^1^H-NMR (200 MHz, DMSO-*d*_6_) *δ*: 7.76 (1H, s, H-4), 7.57 (1H, s, H-7), 7.06 (2H, d, *J* = 8.6, H-2′,6′), 6.48 (2H, d, *J* = 8.6, H-3′,5′), 5.65 (2H, s, CH_2_), 5.13 (2H, s, NH_2_), 2.36 (6H, s, 2CH_3_). ^13^C-NMR (DMSO-*d*_6_) *δ*: 149.2 (C), 145.5 (C), 132.5 (2C), 132.2 (C), 128.8 (C), 128.2 (2CH), 124.2 (2CH), 114.3 (2CH), 52.3 (CH_2_), 18.8 (2CH_3_).

### 4-((5,6-Dichloro-1*H*-Benzo[*d*][1,2,3]Triazol-1-yl)methyl)aniline (18)

Compound **18** was obtained in 30% total yield; m.p. 203–206°C; TLC (petroleum ether/ethyl acetate 7/3); R_*f*_ = 0.29; ^1^H-NMR (400 MHz, CDCl_3_) *δ*: 8.45 (1H, s, H-4), 8.32 (1H, s, H-7), 7.11 (2H, d, *J* = 8.4, H-2′,6′), 6.51 (2H, d, *J* = 8.4, H-3′,5′), 5.75 (2H, s, CH_2_), 5.13 (2H, s, NH_2_), ^13^C-NMR (DMSO-*d*_6_) *δ*: 145.7 (C), 144.9 (C), 133.8 (2C), 133.0 (C), 129.5 (2CH), 126.6 (2CH), 125.9 (C), 115.4 (2CH), 52.3 (CH_2_).

#### General Procedure to Obtain Final Derivatives 3a-g and 4a-d,f; 5e-g and 6f; 7a-g and 10a-g; 8a-g and 11a-g; 9a-g and 12a-d,f

To a solution of 0.3 g (1.19 mmol) of intermediates **13**, **15**, or **16–18** and Triethylamine 0.17 ml (1.19 mmol) dissolved in 9 ml of DMF, the appropriate acyl dichloride **14a-g** (1.44 mmol, ratio 1:1.2) was added. The mixture was left to react at r.t. under stirring for a varying time from 3 to 96 h (3 h for compounds **3g**, **5e**, **7a**-**g**, **9a**,**e**; 9 h for compounds **3b**, **9b**,**c**; 24 h for compounds **3c**,**e**, **5g**, **8a**-**e**, **9f**,**g**; 48 h for compounds **5f**, **9d**; 72 h for compounds **3a**,**d**,**f**; 96 h for compounds **8f**,**g**). Then, Triethylamine hydrochloride was removed by filtration with vacuum and the mothers were diluted with water to obtain a precipitate, which consisted of a mixture of the two products: the bis-benzotriazole dicarboxamides (series **3**, **5**, **7**, **8**, **9**) and the corresponding mono-substituted acids (series **4**, **6**, **10**, **11**, **12**). The separation of the two compounds (dimer and corresponding acid) was obtained by flash chromatography using a mixture of chloroform and methanol (ratio 9.8/0.2) as eluent for the pairs **7**/**10** and **8**/**11** in which the dimer derivatives is the first to be eluted and represents only 1/3 of the mixture, while derivatives bearing two chlorine atoms in the benzotriazole moiety (pairs **3/4**, **5/6**, and **9/12**) were separated by repeated washing of the solid on a porous septum with a heated (60°C) mixture of chloroform, acetone and methanol in a 6:3:1 ratio. The mono-substituted acidic derivatives (**4**, **6**, **12**) were solved by the mixture and thereafter recovered by evaporation *in vacuo* of the solvent and further purified by fractional crystallization using ethyl acetate and petroleum ether. The dicarboxamide derivative, instead, remained unsolved onto the filter was further purified by repeated washings with acetone.

### *N* ^*1*^,*N* ^*4*^-Bis(4-(5,6-Dichloro-1*H*-Benzo[*d*][1,2,3]Triazol-1-yl)phenyl)succinamide (3a)

Compound **3a** was obtained in 20% total yield; m.p. 264–265°C; TLC (chloroform/methanol 9.5/0.5); R_*f*_ = 0.84; ^1^H-NMR (400 MHz, DMSO-*d*_6_) *δ*: 10.43 (2H, s, NH), 8.58 (2H, s, H-4), 8.44 (2H, s, H-7), 7.91 (4H, d, *J* = 8.0, H-3′,5′), 7.86 (4H, d, *J* = 8.0, H-2′,6′), 2.78 (4H, s, CH_2_-CH_2_). ^13^C-NMR (DMSO-*d*_6_) *δ*: 170.8 (2CO), 144.6 (C) 143.2 (C), 140.9 (C), 140.1 (2C), 133.9 (C), 131.8 (C), 131.1 (C), 130.3 (2C), 127.6 (2C), 123.6 (4CH), 121.1 (CH), 120.8 (CH), 119.8 (2CH), 119.5 (CH), 119.4 (CH), 112.8 (2CH), 31.2 (CH_2_), 28.7 (CH_2_). C_28_H_18_Cl_4_N_8_O_2_; MW 640.31; Elem. Anal.: Calcd. C 52.52, H 2.83, N 17.50 Found C 52.25, H 3.00, N 17.56. LC/MS: *m/z* 661 [M + Na]^+^, 641 [M + H]^+^, 639 [M + H]^+^.

### 4-((4-(5,6-Dichloro-1*H*-Benzo[*d*][1,2,3]Triazol-1-yl)phenyl)amino)-4-oxobutanoic Acid (4a)

Compound **4a** was obtained in 22% total yield; m.p. 163–164°C; TLC (chloroform/methanol 9.5/0.5); R_*f*_ = 0.24; ^1^H-NMR (400 MHz, DMSO-*d*_6_) *δ*: 10.34 (1H, s, NH), 8.62 (1H, s, H-4), 8.22 (1H, s, H-7), 7.89 (2H, d, *J* = 8.4, H-3′,5′), 7.81 (2H, d, *J* = 8.4, H-2′,6′), 2.59 (2H, t, CH_2_), 2.57 (2H, t, CH_2_). ^13^C-NMR (DMSO-*d*_6_) *δ*: 173.8 (NHCO), 170.6 (COOH), 144.6 (2C), 143.2 (C), 140.9 (C), 140.1 (2C), 135.1 (C), 131.8 (C), 131.1 (C), 130.3 (C), 127.6 (2C), 123.6 (2CH), 120.8 (CH), 120.2 (2CH), 112.8 (CH), 31.1 (CH_2_), 28.7 (CH_2_). C_16_H_12_Cl_2_N_4_O_3_; MW 379.20; Elem. Anal.: Calcd C 50.68, H 3.19, N 14.78 Found C 50.24, H 3.38, N 14.97. LC/MS: *m/z* 417 [M + K]^+^, 381 [M + H]^+^, 379 [M + H]^+^.

### *N* ^*1*^,*N* ^*5*^-Bis(4-(5,6-Dichloro-1*H*-Benzo[*d*][1,2,3]Triazol-1-yl)phenyl)glutaramide (3b)

Compound **3b** was obtained in 22% total yield; m.p. 266–267°C; TLC (chloroform/methanol 9.5/0.5); R_*f*_ = 0.62; ^1^H-NMR (400 MHz, DMSO-*d*_6_) *δ*: 10.30 (2H, s, NH), 8.61 (2H, s, H-4), 8.25 (2H, s, H-7), 7.92 (4H, d, *J* = 8.0, H-3′,5′), 7.82 (4H, d, *J* = 8.0, H-2′,6′), 2.51 (4H, s, 2CH_2_CO), 2.01 (2H, t, -CH_2_-). ^13^C-NMR (DMSO-*d*_6_) *δ*: 171.2 (2CO), 144.6 (2C), 140.1 (2C), 131.8 (2C), 131.1 (2C), 130.3 (2C), 127.6 (2C), 123.6 (4CH), 120.8 (2CH), 119.9 (4CH), 112.8 (2CH), 35.6 (2CH_2_), 20.7 (CH_2_). C_29_H_20_Cl_4_N_8_O_2_; MW 654.33; Elem. Anal.: Calcd C 53.23, H 3.08, N 17.12 Found C 52.93, H 3.35, N 17.28. LC/MS: *m/z* 677 [M + Na]^+^, 657 [M + H]^+^, 653 [M + H]^+^.

### 5-((4-(5,6-Dichloro-1*H*-Benzo[*d*][1,2,3]Triazol-1-yl)phenyl)amino)-5-oxopentanoic Acid (4b)

Compound **4b** was obtained in 10% total yield; m.p. 106–108°C; TLC (chloroform/methanol 9.5/0.5); R_*f*_ = 0.36; ^1^H-NMR (400 MHz, DMSO-*d*_6_) *δ*: 10.56 (1H, s, NH), 8.60 (1H, s, H-4), 8.22 (1H, s, H-7), 7.90 (2H, d, *J* = 8.4, H-3′,5′), 7.81 (2H, d, *J* = 8.4, H-2′,6′), 2.43 (2H, s, CH_2_CONH), 2.31 (2H, s CH_2_COOH), 1.86 (2H, s, CH_2_). ^13^C-NMR (DMSO-*d*_6_) *δ*: 174.1 (CONH), 171.2 (COOH), 144.6 (C), 140.1 (C), 131.8 (C), 131.1 (C), 130.3 (C), 127.6 (C), 123.6 (2CH), 120.8 (CH), 119.9 (2CH), 112.8 (CH), 35.4 (CH_2_), 32.9 (CH_2_), 20.3 (CH_2_). C_17_H_14_Cl_2_N_4_O_3_; MW 393.22; Elem. Anal.: Calcd C 51.93, H 3.59, N 14.25 Found C 52.04, H 3.75, N 14.00. LC/MS: *m/z* 415 [M + Na]^+^, 393 [M + H]^+^.

### *N* ^*1*^ *,N* ^*6*^-Bis(4-(5,6-Dichloro-1*H*-Benzo[*d*][1,2,3]Triazol-1-yl)phenyl)adipamide (3c)

Compound **3c** was obtained in 15% total yield; m.p. 293–294°C; TLC (chloroform/methanol 9.5/0.5); R_*f*_ = 0.62; ^1^H-NMR (400 MHz, DMSO-*d*_6_) *δ*: 10.28 (2H, s, NH), 8.60 (2H, s, H-4), 8.22 (2H, s, H-7), 7.90 (4H, d, *J* = 8.0, H-3′,5′), 7.80 (4H, d, *J* = 8.0, H-2′,6′), 2.44 (4H, s, 2CH_2_CO), 1.23 (4H, s, CH_2_-CH_2_). ^13^C-NMR (DMSO-*d*_6_) *δ*: 171.5 (2CO) 144.6 (C), 143.2 (C), 140.9 (C), 140.1 (2C), 134.0 (C), 131.8 (C), 131.1 (C), 130.3 (2C), 127.6 (2C), 123.6 (4CH), 121.1 (CH), 120.8 (CH), 119.9 (2CH), 119.7 (CH), 119.4 (CH), 112.8 (2CH), 36.3 (2CH_2_), 24.8 (2CH_2_). C_30_H_22_Cl_4_N_8_O_2_; MW 668.36; Elem. Anal.: Calcd C 53.91, H 3.32, N 16.77 Found C 54.22, H 3.34, N 16.45. LC/M: *m/z* 671 [M + H]^+^, 669 [M + H]^+^, 667 [M + H]^+^.

### 6-((4-(5,6-Dichloro-1*H*-Benzo[*d*][1,2,3]Triazol-1-yl)phenyl)amino)-6-oxohexanoic Acid (4c)

Compound **4c** was obtained in 10% total yield; m.p. 148–150°C; TLC (chloroform/methanol 9.5/0.5); R_*f*_ = 0.26; ^1^H-NMR (400 MHz, DMSO-*d*_6_) *δ*: 10.26 (1H, s, NH), 8.61 (1H, s, H-4), 8.24 (1H, s, H-7), 7.90 (2H, d, *J* = 8.0, H-3′,5′), 7.81 (2H, d, *J* = 8.0, H-2′,6′), 2.38 (2H, t, CH_2_CONH), 2.27 (2H, t, CH_2_COOH), 1.70–1.56 (4H, m, CH_2_-CH_2_). ^13^C-NMR (DMSO-*d*_6_) *δ*: 174.3 (CONH), 171.5 (COOH), 144.6 (C), 140.1 (C), 131.8 (C), 131.1 (C), 130.3 (C), 127.6 (C), 123.6 (2CH), 120.8 (CH), 119.9 (2CH), 112.8 (CH). C_18_H_16_Cl_2_N_4_O_3_; MW 407.25; Elem. Anal.: Calcd C 53.09, H 3.96, N 13.76 Found C 52.79, H 4.26, N 14.08. LC/MS: *m/z* 429 [M + Na]^+^, 411 [M + H]^+^, 409 [M + H]^+^, 407 [M + H]^+^.

### *N* ^*1*^ *,N* ^*4*^-Bis(4-(5,6-Dichloro-1*H*-Benzo[*d*][1,2,3]Triazol-1-yl)phenyl)fumaramide (3d)

Compound **3d** was obtained in 18% total yield; m.p. >300°C; TLC (chloroform/methanol 9.5/0.5); R_*f*_ = 0.90; ^1^H-NMR (400 MHz, DMSO-*d*_6_) *δ*: 10.92 (1H, s, NH), 8.64 (2H, s, H-4), 8.32 (2H, s, H-7), 8.02 (4H, d, *J* = 8.4, H-3′,5′), 7.91 (4H, d, *J* = 8.4, H-2′,6′), 7.31 (2H, s, CH=CH). ^13^C-NMR (DMSO-*d*_6_) *δ*: 162.3 (2CO), 144.7 (2C), 141.5 (2C), 139.5 (2C), 134.8 (2C), 134.2 (CH=CH), 131.1 (2C), 127.7 (2C), 123.8 (4CH), 120.9 (2CH), 120.5 (4CH), 112.9 (2CH). C_28_H_16_Cl_4_N_8_O_2_; MW 638.29; Elem. Anal.: Calcd C 52.69, H 2.53, N 17.56 Found C 52.59, H 2.75, N 17.22. LC/MS: *m/z* 659 [M + Na]^+^, 641 [M + H]^+^, 637 [M + H]^+^.

### (*E*)-4-((4-(5,6-Dichloro-1*H*-Benzo[*d*][1,2,3]Triazol-1-yl)phenyl)amino)-4-oxobut-2-enoic Acid (4d)

Compound **4d** was obtained in 20% total yield; m.p. 231–233°C; TLC (chloroform/methanol 9.5/0.5); R_*f*_ = 0.15; ^1^H-NMR (400 MHz, DMSO-*d*_6_) *δ*: 10.99 (1H, s, NH), 8.63 (1H, s, H-4), 8.35 (1H, s, H-7), 8.09 (2H, d, *J* = 8.0, H-3′,5′), 7.92 (2H, d, *J* = 8.0, H-2′,6′), 7.31 (1H, d, *J* = 15.2, =CH-COOH), 6.78 (1H, d, *J* = 15.2, NHCO-CH=). ^13^C-NMR (DMSO-*d*_6_) *δ*: 166.2 (CONH), 162.0 (COOH), 144.6 (C), 139.3 (C), 136.8 (HNOC-CH=), 131.8 (C), 131.2 (=CH-COOH), 131.1 (C), 130.5 (C), 127.7 (C), 123.7 (2CH), 120.9 (CH) 120.5 (2CH), 112.9 (CH). C_16_H_10_Cl_2_N_4_O_3_; MW 377.18; Elem. Anal.: Calcd C 50.95, H 2.67, N 14.85 Found C 51.00, H 2.44, N 15.10. LC/MS: *m/z* 379 [M + H]^+^, 377 [M + H]^+^.

### *N* ^*1*^,*N* ^*4*^-Bis(4-(5,6-Dichloro-1*H*-Benzo[*d*][1,2,3]Triazol-1-yl)phenyl)terephthalamide (3e)

Compound **3e** was obtained in 17% total yield; m.p. 197–199°C; TLC (chloroform/methanol 9/1); R_*f*_ = 0.81; ^1^H-NMR (400 MHz, DMSO-*d*_6_) *δ*: 10.76 (2H, s, NH), 8.63 (2H, s, 2H-4), 8.31 (2H, s, 2H-7), 8.12–8.04 (6H, m, H-2″,3″,5″,6″,2′,6′), 8.02 (2H, m, H-2′,6′), 7.91 (4H, m, 2H-3′,5′). ^13^C-NMR (DMSO-*d*_6_) *δ*: 166.7 (2CO), 144.7 (2C), 139.8 (2C), 138.3 (2C), 134.5 (2C), 131.9 (2C), 131.1 (2C), 129.4 (8CH), 128.0 (2CH), 127.7 (2C), 123.5 (4CH), 121.3 (2CH). C_32_H_18_Cl_4_N_8_O_2_; MW 688.34; Elem. Anal.: Calcd C 55.84, H 2.64, N 16.28 Found C 55.42, H 2.91, N 15.98. LC/MS: *m/z* 689 [M + H]^+^, 687 [M + H]^+^.

### *N* ^*2*^,*N* ^*6*^-Bis(4-(5,6-Dichloro-1*H*-Benzo[*d*][1,2,3]Triazol-1-yl)phenyl)pyridine-2,6-dicarboxamide (3f)

Compound **3f** was obtained in 6% total yield; m.p. >300°C; TLC (chloroform/methanol 9/1); R_*f*_ = 0.8; ^1^H-NMR (400 MHz, DMSO-*d*_6_) *δ*: 11.35 (2H, s, NH), 8.65 (2H, s, H-4), 8.52–8.48 (3H, m, 2H-7, H-4″), 8.43–8.30 (6H, m, 2H-2′, 2H-6′, H-3″, H-5″), 8.04–8.02 (4H, m, 2H-3′,2H-5′). ^13^C-NMR (DMSO-*d*_6_) *δ*: 162.0 (2CO), 148.6 (2C), 144.7 (2C), 143.4 (C), 139.9 (C), 138.9 (2C), 132.0 (2C), 131.6 (C), 131.1 (C), 130.6 (C), 127.8 (C), 125.7 (CH), 123.6 (4CH), 121.8 (4CH), 121.2 (CH), 120.9 (CH), 119.5 (2CH), 113.0 (2CH). C_31_H_17_Cl_4_N_9_O_2_; MW 689.33; Elem. Anal.: Calcd C 54.02, H 2.49, N 16.28 Found C 54.38, H 2.50, N 16.45. LC/MS: *m/z* 690 [M + H]^+^, 692 [M + H]^+^.

### 6-((4-(5,6-Dichloro-1*H*-Benzo[*d*][1,2,3]Triazol-1-yl)phenyl)carbamoyl)picolinic Acid (4f)

Compound **4f** was obtained in 30% total yield; m.p. 240°C; TLC (chloroform/methanol 9/1); R_*f*_ = 0.1; ^1^H-NMR (400 MHz, DMSO-*d*_6_) *δ*: 11.39 (1H, s, OH), 11.16 (1H, s, NH), 8.80 (1H, s, H-4), 8.58 (1H, t, H-4″), 8.31 (5H, s, H-7, H-2′,3′,5′,6′), 8.16 (1H, d, *J* = 8.0, H-3″), 7.98 (1H, d, *J* = 8.0, H-5″). ^13^C-NMR (DMSO-*d*_6_) *δ*: 164.9 (CO), 161.9 (CO), 148.9 (C), 146.7 (C), 144.7 (C), 140.1 (CH), 138.8 (C), 131.9 (C), 131.5 (C), 131.0 (C), 127.7 (C), 127.2 (CH), 125.8 (CH), 123.5 (CH), 123.4 (CH), 121.8 (CH), 121.6 1CH), 120.9 (CH), 113.0 (CH). C_19_H_11_Cl_2_N_5_O_3_; MW 428.22; Elem. Anal.: Calcd C 53.29, H 2.59, N 16.56 Found C 53.39, H 2.79, N 16.25. LC/MS: *m/z* 429 [M + H]^+^, 427 [M + H]^+^.

### *N* ^*2*^,*N* ^*5*^-Bis(4-(5,6-Dichloro-1*H*-Benzo[*d*][1,2,3]Triazol-1-yl)phenyl)thiophene-2,5-dicarboxamide (3g)

Compound **3g** was obtained in 15% total yield; m.p. 208–209°C; TLC (chloroform/methanol 9/1); R_*f*_ = 0.87; ^1^H-NMR (400 MHz, DMSO-*d*_6_) *δ*: 10.75 (2H, s, NH), 8.64 (2H, s, 2H-4), 8.34 (2H, s, 2H-7), 8.12 (2H, d, *J* = 8.01, H-3″,4″), 8.08 (4H, s, 2H-2′,6′), 7.93 (4H, s, 2H-3′,5′). ^13^C-NMR (DMSO-*d*_6_) *δ*: 159.6 (CO), 159.5 (CO), 144.7 (2C), 143.8 (2C), 139.3 (2C), 131.9 (2C), 131.3 (2C), 131.1 (2C), 129.8 (2CH), 127.7 (2C), 123.6 (4CH), 121.4 (4CH), 120.9 (2CH), 112.9 (2CH). C_30_H_16_Cl_4_N_8_O_2_S; MW 694.37; Elem. Anal.: Calcd C 51.90, H 2.32, N 16.14 Found C 51.90, H 2.32, N 16.14. LC/MS: *m/z* 495 [M + H]^+^, 493 [M + H]^+^.

### *N* ^*1*^,*N* ^*4*^-Bis(4-(5,6-Dichloro-2*H*-Benzo[*d*][1,2,3]Triazol-2-yl)phenyl)terephthalamide (5e)

Compound **5e** was obtained in 30% total yield; m.p. >300°C; TLC (chloroform/methanol 9/1); R_*f*_ = 0.64; ^1^H-NMR (400 MHz, DMSO-*d*_6_) *δ*: 10.77 (2H, s, NH), 8.50 (4H, s, 2H-4, 2H-7), 8.31 (4H, d, *J* = 8.0, 2H-2′, 2H-6′), 8.12–8.09 (6H, m, 2H-3′,2H-5′, H-2″,6″), 8.04 (2H, s, H-3″,5″). ^13^C-NMR (DMSO-*d*_6_) *δ*: 166.6 (CO), 165.2 (CO), 143.3 (4C), 140.6 (2C), 138.3 (2C), 134.7 (C), 134.4 (2C), 133.5 (C), 130.5 (2C), 129.4 (4CH), 129.3 (2CH), 128.0 (2CH), 121.0 (4CH), 119.5 (4CH). C_32_H_18_Cl_4_N_8_O_2_; MW 688.34; Elem. Anal.: Calcd C 55.84, H 2.64, N 18.28 Found C 55.56, H 2.34, N 18.59. LC/MS: *m/z* 689 [M + H]^+^, 687 [M + H]^+^.

### *N* ^*2*^,*N* ^*6*^-Bis(4-(5,6-Dichloro-2*H*-Benzo[*d*][1,2,3]Triazol-2-yl)phenyl)pyridine-2,6-dicarboxamide (5f)

Compound **5f** was obtained in 13% total yield; m.p. >300°C; TLC (chloroform/methanol 9/1); R_*f*_ = 0.20; ^1^H-NMR (400 MHz, DMSO-*d*_6_) *δ*: 11.16 (2H, s, NH), 8.51 (4H, s, 2H-4, 2H-7), 8.45–8.34 (7H, m, H-3″,4″,5″,2′,6′), 8.16 (4H, d, *J* = 8.8, 2H-3′,5′). ^13^C-NMR (DMSO-*d*_6_) *δ*: 150.8 (2CO), 143.3 (2C), 143.0 (4C), 130.5 (2C), 129.4 (4C), 128.3 (2C), 121.7 (4CH), 121.2 (1CH), 119.5 (2CH), 118.9 (4CH), 113.7 (4CH). C_31_H_17_Cl_4_N_9_O_2_; MW 689.33; Elem. Anal.: Calcd C 54.02, H 2.49, N 16.28 Found C 53.70, H 2.19, N 16.58. LC/MS: *m/z* 690 [M + H]^+^, 688 [M + H]^+^.

### 6-((4-(5,6-Dichloro-2*H*-Benzo[*d*][1,2,3]Triazol-2-yl)phenyl)carbamoyl)picolinic Acid (6f)

Compound **6f** was obtained in 30% total yield; m.p. 240°C; TLC (chloroform/methanol 9/1); R_*f*_ = 0.1; ^1^H-NMR (400 MHz, DMSO-*d*_6_) *δ*: 11.39 (1H, s, OH), 11.16 (1H, s, NH), 8.80 (1H, s, H-4), 8.58 (1H, t, H-4″), 8.31 (5H, s, H-7, H-2′,3′,5′,6′), 8.16 (1H, d, *J* = 8.0, H-3″), 7.98 (1H, d, *J* = 8.0, H-5″). ^13^C-NMR (DMSO-*d*_6_) *δ*: 164.9 (CO), 161.9 (CO), 148.9 (C), 146.7 (C), 144.7 (C), 140.1 (CH), 138.8 (C), 131.9 (C), 131.5 (C), 131.0 (C), 127.7 (C), 127.2 (CH), 125.8 (CH), 123.5 (CH), 123.4 (CH), 121.8 (CH), 121.6 (CH), 120.9 (CH), 113.0 (CH). C_19_H_11_Cl_2_N_5_O_3_; MW 428.22; Elem. Anal.: Calcd C 53.29, H 2.59, N 16.56 Found C 53.59, H 2.72, N 16.64. LC/MS: *m/z* 429 [M + H]^+^, 428 [M + H]^+^.

### *N* ^*2*^,*N* ^*5*^-Bis(4-(5,6-Dichloro-2*H*-Benzo[*d*][1,2,3]Triazol-2-yl)phenyl)thiophene-2,5-Dicarboxamide (5g)

Compound **5g** was obtained in 11% total yield; m.p. >300°C; TLC (chloroform/methanol 9/1); R_*f*_ = 0.74; ^1^H-NMR (400 MHz, DMSO-*d*_6_) *δ*: 10.73 (2H, s, NH), 8.51 (4H, s, 2H-4, 2H-7), 8.32 (4H, d, *J* = 8.8, 2H-2′,6′), 8.06 (6H, m, 2H-3′,5′, H-3″,4″). ^13^C-NMR (DMSO-*d*_6_) *δ*: 162.5 (CO), 159.7 (CO), 143.3 (4C), 140.1 (4C), 134.8 (2C), 130.5 (4C), 129.8 (2CH), 121.1 (8CH), 119.5 (4CH). C_30_H_16_Cl_4_N_8_O_2_; MW 694.37; Elem. Anal.: Calcd C 51.90, H 2.32, N 16.14 Found C 52.00, H 2.55, N 15.90. LC/MS: *m/z* 695 [M + H]^+^, 697 [M + H]^+^.

### *N* ^*1*^,*N* ^*4*^-Bis(4-((1*H*-Benzo[*d*][1,2,3]Triazol-1-yl)methyl)phenyl)succinamide (7a)

Compound **7a** was obtained in 10% total yield; m.p. 255–257°C; TLC (chloroform/methanol 9.5/0.5); R_*f*_ = 0.55; ^1^H-NMR (200 MHz, DMSO-*d*_6_) *δ*: 10.04 (2H, s, 2NH), 8.04 (2H, d, *J* = 8.2, 2H-4), 7.81 (2H, d, *J* = 8.2, 2H-7), 7.53 (4H, d, *J* = 8.4, 2H-2′, 2H-6′), 7.51–7.48 (2H, m, 2H-5) 7.39 (2H, t, H-6) 7.27 (4H, d, *J* = 8.4, 2H-3′,2H-5′), 5.90 (4H, s, 2CH_2_), 2.59 (4H, s, 2CH_2_). ^13^C-NMR (DMSO-*d*_6_) *δ*: 170.4 (2CO), 145.3 (2C), 139.1 (2C), 132.5 (2C), 130.0 (2C), 128.4 (4CH), 127.3 (2CH), 123.9 (2CH), 119.2 (2CH), 119.1 (4CH), 110.7 (2CH), 50.7 (2CH_2_), 31.1 (2CH_2_). C_30_H_26_N_8_O_2_; MW 530.58; Elem. Anal.: Calcd C 67.91, H 4.94, N 21.12 Found C 68.00, H 4.55, N 16.35. LC/MS *m/z* 531 [M + H]^+^.

### 4-((4-((1*H*-Benzo[*d*][1,2,3]Triazol-1-yl)methyl)phenyl)amino)-4-Oxobutanoic Acid (10a)

Compound **10a** was obtained in 10% total yield; m.p. 193–195 °C; TLC (chloroform/methanol 9.5/0.5); R_*f*_ = 0.15; ^1^H-NMR (200 MHz, DMSO-*d*_6_) *δ*: 10.04 (H, s, NH), 8.02 (H, d, *J* = 8.2, H-4), 7.81 (H, d, *J* = 8.2, H-7), 7.62–7.50 (3H, m, H-2′,6′ and H-5), 7.40 (H, t, H-6) 7.31 (2H, d, *J* = 9.2, H-3′,5′), 5.90 (H, s, CH_2_), 2.53 (4H, s, 2CH_2_). ^13^C-NMR (DMSO-*d*_6_) *δ*: 173.7 (CO), 170.1 (CO), 145.3 (C), 139.1 (C), 132.5 (C), 130.0 (C), 128.4 (2CH), 127.3 (CH), 124.0 (CH), 119.2 (CH), 119.1 (2CH), 110.7 (CH), 50.7 (CH_2_), 31.1 (CH_2_), 28.7 (CH_2_). C_17_H_16_N_4_O_3_; MW 324.33; Elem. Anal.: Calcd C 62.95, H 4.97, N 17.27 Found C 62.65, H 4.78, N 17.54. LC/MS *m/z* 325 [M + H]^+^.

### *N* ^*1*^,*N* ^*5*^-Bis(4-((1*H*-benzo[*d*][1,2,3]Triazol-1-yl)methyl)phenyl)GlutAramide (7b)

Compound **7b** was obtained in 12% total yield; m.p. 237–238 °C; TLC (chloroform/methanol 9.5/0.5); R_*f*_ = 0.46; ^1^H-NMR (200 MHz, DMSO-*d*_6_) *δ*: 9.94 (2H, s, 2NH), 8.06 (2H, d, *J* = 8.2, 2H-4), 7.82 (2H, d, *J* = 8.6, H-7), 7.60–7.48 (6H, m, 2H-2′,6′ and 2H-5), 7.39 (2H, t, 2H-6), 7.28 (4H, d, *J* = 8.4, 2H-3′,5′), 5.90 (4H, s, 2CH_2_), 2.31 (4H, t, 2CH_2_CO), 1.84 (2H, m, CH_2_ CH_2_CH_2_). ^13^C-NMR (DMSO-*d*_6_) *δ*: 170.8 (2CO), 145.3 (2C), 139.1 (2C), 132.5 (2C), 130.1 (2C), 128.7 (2CH), 128.4 (2CH), 127.3 (2CH), 123.8 (2CH), 119.6 (2CH), 119.2 (2CH), 110.7 (2CH), 110.4 (2CH), 50.7 (2CH_2_), 35.5 (2CH_2_), 20.8 (CH_2_). C_31_H_28_N_8_O_2_; MW 544.61; Elem. Anal.: Calcd C 68.37, H 5.18, N 20.58 Found C 68.64, H 5.18, N 20.80. LC/MS *m/z* 545 [M + H]^+^.

### 5-((4-((1*H*-Benzo[*d*][1,2,3]Triazol-1-yl)methyl)phenyl)amino)-5-Oxopentanoic Acid (10b)

Compound **10b** was obtained in 25% total yield; m.p. 152–154°C; TLC (chloroform/methanol 9.5/0.5); R_*f*_ = 0.20; ^1^H-NMR (200 MHz, DMSO-d_6_) *δ*: 9.99 (1H, s, NH), 8.04 (1H, d, *J* = 7.6, H-4), 7.82 (1H, d, *J* = 7.0, H-7), 7.56–7.42 (3H, m, H-2′,6′, H-5), 7.38 (1H, t, H-6), 7.28 (2H, d, *J* = 8.4, H-3′,5′), 5.90 (2H, s, CH_2_), 2.31 (2H, t, CH_2_CO), 2.22 (2H, t, CH_2_CO), 1.77 (2H, m, CH_2_CH_2_CH_2_). ^13^C-NMR (DMSO-*d*_6_) *δ*: 174.5 (CO), 170.9 (CO), 145.3 (C), 139.1 (C), 132.5 (C), 130.1 (C), 128.6 (2CH), 128.3 (CH), 124.0 (CH), 119.2 (CH), 119.1 (2CH), 110.7 (CH), 50.7 (CH_2_), 35.4 (2CH_2_), 20.5 (CH_2_). C_18_H_18_N_4_O_3_; MW 338.36; Elem. Anal.: Calcd C 63.89, H 5.36, N 16.56 Found C 63.53, H 5.26, N 16.64. LC/MS *m/z* 339 [M + H]^+^.

### *N* ^*1*^,*N* ^*6*^-Bis(4-((1*H*-Benzo[*d*][1,2,3]Triazol-1-yl)methyl)phenyl)Adipamide (7c)

Compound **7c** was obtained in 16% total yield; m.p. 231–232°C; TLC (chloroform/methanol 9.5/0.5); R_*f*_ = 0.39; ^1^H-NMR (200 MHz, DMSO-*d*_6_) *δ*: 9.91 (2H, s, 2NH), 8.03 (2H, d, *J* = 8.0, 2H-4), 7.80 (2H, d, *J* = 8.2, 2H-7), 7.58–7.41 (6H, m, 2H-2′,6′, 2H-5), 7.38 (2H, t, 2H-7), 7.27 (4H, d, *J* = 8.6, 2H-3′,5′), 5.90 (4H, s, 2CH_2_), 2.27 (4H, t, 2CH_2_CO), 1.54 (4H, m, CH_2_CH_2_). ^13^C-NMR (DMSO-*d*_6_) *δ*: 171.6 (2CO), 145.8 (2C), 139.6 (2C), 133.0 (2C), 130.6 (2C), 128.8 (4CH), 127.8 (2CH), 124.4 (2CH), 119.7 (2CH), 119.6 (4CH), 111.2 (2CH), 51.2 (2CH_2_), 36.6 (2CH_2_), 25.2 (2CH_2_). C_32_H_30_N_8_O_2_; MW 558.63; Elem. Anal.: Calcd C 68.80, H 5.41, N 20.06 Found C 68.44, H 5.61, N 20.26. LC/MS *m/z* 559 [M + H]^+^.

### 6-((4-((1*H*-Benzo[*d*][1,2,3]triazol-1-yl)methyl)phenyl)amino)-6-oxohexanoic Acid (10c)

Compound **10c** was obtained in 33% total yield; m.p. 134–136 °C; TLC (chloroform/methanol 9.5/0.5); R_*f*_ = 0.23; ^1^H-NMR (200 MHz, DMSO-*d*_6_) *δ*: 9.93 (1H, s, NH), 8.04 (1H, d, *J* = 8.2, H-4), 7.82 (1H, d, *J* = 8.2 H-7), 7.57–7.48 (3H, m, H-2′,6′, H-5), 7.39 (1H, t, H-7), 7.29 (2H, d, *J* = 8.2, H-3′,5′), 5.90 (2H, s, CH_2_), 2.27 (2H, t, CH_2_CO), 2.21 (2H, m, CH_2_CO), 1.54 (4H, m, CH_2_CH_2_). ^13^C-NMR (DMSO-*d*_6_) *δ*: 174.3 (CO), 171.1 (CO), 145.3 (C), 139.1 (C), 132.5 (C), 130.1 (C), 128.4 (2CH), 127.3 (CH), 124.0 (CH), 119.2 (CH), 119.2 (2CH), 110.7 (CH), 50.7 (CH_2_), 36.0 (CH_2_), 33.4 (CH_2_), 24.6 (CH_2_), 24.1 (CH_2_). C_19_H_20_N_4_O_3_; MW 352.39; Elem. Anal.: Calcd C 64.76, H 5.72, N 15.90 Found C 65.00, H 5.93, N 16.00. LC/MS: *m/z* 353 [M + H]^+^.

### *N* ^*1*^,*N* ^*4*^-Bis(4-((1*H*-Benzo[*d*][1,2,3]Triazol-1-yl)methyl)phenyl)Fumaramide (7d)

Compound **7d** was obtained in 12% total yield; m.p. 268–269°C; TLC (chloroform/methanol 9.5/0.5); R_*f*_ = 0.41; ^1^H-NMR (200 MHz, DMSO-*d*_6_) *δ*: 10.58 (2H, s, 2NH), 8.04 (2H, d, *J* = 8.2, 2H-4), 7.83 (2H, d, *J* = 8.2, 2H-7), 7.65 (4H, d, *J* = 8.2, 2H-2′,6′), 7.53 (2H, t, 2H-5) 7.43 (2H, t, 2H-6) 7.34 (4H, d, *J* = 8.4, 2H-3′,5′), 7.14 (2H, s, CH = CH), 5.94 (4H, s, 2CH_2_). ^13^C-NMR (DMSO-*d*_6_) *δ*: 162.1 (2CO), 143.3 (2C), 138.6 (2C), 134.0 (2CH), 132.5 (2C), 131.1 (2C), 128.5 (2CH), 128.4 (2CH), 127.4 (2CH), 124.0 (2CH), 119.6 (2CH), 119.5 (2CH), 119.2 (2CH), 110.7 (2CH), 50.7 (2CH_2_). C_30_H_24_N_8_O_2_; MW 528.56; Elem. Anal.: Calcd C 64.76, H 5.72, N 15.90 Found C 64.33, H 5.54, N 16.20. LC/MS: *m/z* 353 [M + H]^+^.

### (*E*)-4-((4-((1*H*-Benzo[*d*][1,2,3]Triazol-1-yl)methyl)phenyl)amino)-4-oxobut-2-enoic Acid (10d)

Compound **10d** was obtained in 30% total yield; m.p. 290–293°C; TLC (chloroform/methanol 9.5/0.5); R_*f*_ = 0.11; ^1^H-NMR (200 MHz, DMSO-*d*_6_) *δ*: 10.22 (1H, s, NH), 8.04 (1H, d, *J* = 7.6, H-4), 7.82 (1H, d, *J* = 9.0, H-7), 7.63 (2H, d, *J* = 8.2, H-2′,6′), 7.52 (1H, t, H-5) 7.42 (1H, t, H-6) 7.30 (2H, d, *J* = 8.2, H-3′,5′), 6.61 (2H, s, CH = CH), 5.91 (2H, s, CH_2_). ^13^C-NMR (DMSO-*d*_6_) *δ*: 168.1 (CO), 164.0 (CO), 145.4 (C), 139.1 (C), 134.0 (2CH), 132.5 (C), 130.5 (C), 130.0 (CH), 128.4 (2CH), 127.3 (CH), 124.0 (CH), 119.6 (CH), 119.4 (CH), 119.2 (CH), 110.7 (CH), 50.7 (CH_2_). C_17_H_14_N_4_O_3_; MW 322.32; Elem. Anal.: Calcd C 63.35, H 4.38, N 17.38 Found C 63.35, H 4.40, N 17.25. LC/MS: *m/z* 353 [M + H]^+^.

### *N* ^*1*^,*N* ^*4*^-Bis(4-((1*H*-Benzo[*d*][1,2,3]Triazol-1-yl)methyl)phenyl)Terephthalamide (7e)

Compound **7e** was obtained in 15% total yield; m.p. >300°C; TLC (chloroform/methanol 9.5/0.5); R_*f*_ = 0.43; ^1^H-NMR (200 MHz, DMSO-*d*_6_) *δ*: 10.44 (2H, s, 2NH), 8.04–8.01 (4H, m, H-2″,3″,5″,6″), 7.87–7.82 (4H, m, 4H_arom_), 7.75 (4H, d, *J* = 8.4, 2H-2′,6′), 7.60–7.42 (4H, m, 4H_arom_), 7.36 (4H, d, *J* = 8.4, 2H-3′,5′), 5.95 (4H, s, 2CH_2_). ^13^C-NMR (DMSO-*d*_6_) *δ*: 164.8 (2CO), 145.4 (2C), 138.8 (2C), 138.5 (C), 133.4 (C), 132.6 (2C), 131.1 (2C), 129.4 (2CH), 129.2 (2CH), 128.3 (4CH), 127.9 (4CH), 127.4 (CH), 124.0 (CH), 120.6 (4CH), 119.2 (CH), 110.7 (CH), 50.7 (2CH_2_). C_34_H_26_N_8_O_2_; MW 578.62; Elem. Anal.: Calcd C 70.58, H 4.53, N 19.37 Found C 70.43, H 4.88, N 19.39. LC/MS: *m/z* 579 [M + H]^+^.

### 4-(4-((1*H*-Benzo[*d*][1,2,3]Triazol-1-yl)methyl)phenylcarbamoyl)Benzoic Acid (10e)

Compound **10e** was obtained in 30% total yield; m.p. >300°C; TLC (chloroform/methanol 9.5/0.5); R_*f*_ = 0.12; ^1^H-NMR (200 MHz, DMSO-*d*_6_) *δ*: 10.44 (H, s, NH), 8.06–8.00 (4H, m, H-2″,3″,5″,6″), 7.85 (1H, d, *J* = 8.0, 1H_arom_), 7.78 (2H, d, *J* = 8.4, H-2′,6′), 7.54 (1H, t, *J* = 7.2, 1H_arom_), 7.42–7.40 (2H, m, 2H_arom_), 7.36 (2H, d, *J* = 8.4, H-3′,5′), 5.95 (2H, s, CH_2_). ^13^C-NMR (DMSO-*d*_6_) *δ*: 167.8 (CO), 165.4 (CO), 145.4 (C), 138.9 (C), 137.0 (2C), 132.6 (C), 131.5 (C), 129.1 (CH), 127.5 (CH), 127.4 (CH), 126.7 (CH), 126.5 (2CH), 124.0 (CH), 120.7 (2CH), 120.6 (CH), 117.8 (CH), 110.7 (CH), 50.7 (CH_2_). C_21_H_16_N_4_O_3_; MW 372,38; Elem. Anal.: Calcd C 67.73, H 4.33, N 15.05 Found C 67.63, H 4.22, N 15.35. LC/MS: *m/z* 373 [M + H]^+^.

### *N* ^*2*^,*N* ^*6*^-Bis(4-((1*H*-Benzo[*d*][1,2,3]Triazol-1-yl)methyl)phenyl)Pyridine-2,6-dicarboxamide (7f)

Compound **7f** was obtained in 25% total yield; m.p. 111–112 °C; TLC (chloroform/methanol 9.5/0.5); R_*f*_ = 0.50; ^1^H-NMR (200 MHz, DMSO-*d*_6_) *δ*: 11.00 (2H, s, 2NH), 8.38–8.28 (3H, m, H-3″,4″,5″), 8.06 (4H, d, *J* = 8.2, 4H_arom_), 7.86 (4H, d, *J* = 7.8, 2H-2′,6′), 7.57–7.50 (4H, m, 4H_arom_), 7.42 (4H, d, *J* = 7.8, 2H-3′,5′), 5.99 (4H, s, 2CH_2_). ^13^C-NMR (DMSO-*d*_6_) *δ*: 161.6 (2CO), 148.7 (2C), 145.4 (2C), 140.0 (CH), 137.8 (2C), 132.6 (2C), 131.7 (2C), 128.4 (4CH), 127.4 (2CH), 125.3 (2CH), 124.0 (2CH), 121.3 (4CH), 119.2 (2CH), 110.7 (2CH), 50.7 (2CH_2_). C_33_H_25_N_9_O_2_; MW 579.61; Elem. Anal.: Calcd C 68.38, H 4.35, N 21.75 Found C 68.40, H 4.35, N 22.00. LC/MS: *m/z* 580 [M + H]^+^.

### 6-(4-((1*H*-Benzo[*d*][1,2,3]Triazol-1-yl)methyl)phenylcarbamoyl)Picolinic Acid (10f)

Compound **10f** was obtained in 30% total yield; m.p. 230–232 °C; TLC (chloroform/methanol 9.5/0.5); R_*f*_ = 0.10; ^1^H-NMR (200 MHz, DMSO-*d*_6_) *δ*: 11.00 (H, s, NH), 8.40–8.32 (3H, m, H-3″,4″,5″), 8.06 (2H, d, *J* = 8.2, 2H_arom_), 7.86 (2H, d, *J* = 7.8, H-2′,6′), 7.57–7.50 (2H, m, 2H_arom_), 7.42 (H, d, *J* = 7.8, H-3′,5′), 5.99 (2H, s, CH_2_). ^13^C-NMR (DMSO-*d*_6_) *δ*: 166.8 (CO), 162.8 (CO), 153.2 (C), 148.9 (C), 145.4 (2C), 138.4 (C), 132.6 (C), 127.4 (2CH), 124.0 (2CH), 123.2 (CH), 120.6 (2CH), 119.2 (2CH), 110.7 (2CH), 50.7 (CH_2_). C_20_H_15_N_5_O_3_; MW 373.36; Elem. Anal.: Calcd C 64.34, H 4.05, N 18.76 Found C 64.34, H 4.35, N 18.80 LC/MS: *m/z* 374 [M + H]^+^.

### *N* ^*2*^,*N* ^*5*^-Bis(4-((1*H*-Benzo[*d*][1,2,3]Triazol-1-yl)methyl)phenyl)Thiophene-2,5-Dicarboxamide (7g)

Compound **7g** was obtained in 20% total yield; m.p. >300 °C; TLC (chloroform/methanol 9.5/0.5); R_*f*_ = 0.31; ^1^H-NMR (200 MHz, DMSO-*d*_6_) *δ*: 10.27 (2H, s, 2NH), 8.15–8.07 (2H, d, *J* = 8.4, H-3″,4″), 7.80–7.76 (4H, m, 4H_arom_), 7.64 (4H, d, *J* = 8.2, 2H-2′,6′), 7.46 (2H, t, *J* = 7.2, 2H_arom_), 7.32 (2H, t, *J* = 7.2, 2H_arom_), 7.70 (4H, d, *J* = 8.2, 2H-3′,5′), 5.94 (4H, s, 2CH_2_). ^13^C-NMR (DMSO-*d*_6_) *δ*: 160.1 (2CO), 145.3 (2C), 140.9 (2C), 138.6 (2C), 132.6 (2C), 130.9 (2C), 129.5 (2CH), 128.3 (4CH), 127.4 (2CH), 124.0 (2CH), 120.6 (2CH), 120.5 (2CH), 119.2 (2CH), 110.7 (2CH), 50.7 (2CH_2_). C_32_H_24_N_8_O_2_S; MW 584.65; Elem. Anal.: Calcd C 65.74, H 4.14, N 19.17 Found C 65.44, H 4.24, N 19.32 LC/MS: *m/z* 585 [M + H]^+^.

### 5-(4-((1*H*-Benzo[*d*][1,2,3]Triazol-1-yl)methyl)phenylcarbamoyl)thiophene-2-Carboxylic Acid (10g)

Compound **10g** was obtained in 25% total yield; m.p. >300°C; TLC (chloroform/methanol 9.5/0.5); R_*f*_ = 0.10; ^1^H-NMR (200 MHz, DMSO-*d*_6_) *δ*: 10.27 (H, s, NH), 8.06–8.04 (2H, d, *J* = 8.4, H-3″,4″), 7.85 (2H, d, *J* = 8.2, 2H-2′,6′), 7.70 (4H, d, *J* = 8.2, 2H-3′,5′), 7.52 (H, t, *J* = 7.2, H_arom_), 7.40 (H, t, *J* = 7.2, H_arom_), 7.35 (2H, d, *J* = 8.2, 2H_arom_) 5.94 (2H, s, CH_2_). ^13^C-NMR (DMSO-*d*_6_) *δ*: 160.1 (2CO), 145.3 (2C), 140.9 (2C), 138.6 (2C), 132.6 (2C), 130.9 (2C), 129.5 (2CH), 128.3 (4CH), 127.4 (2CH), 124.0 (2CH), 120.6 (2CH), 120.5 (2CH), 119.2 (2CH), 110.7 (2CH), 50.7 (2CH_2_). C_19_H_14_N_4_O_3_S; MW 378.40; Elem. Anal.: Calcd C 60.31, H 3.73, N 14.81 Found C 60.05, H 3.71, N 14.65 LC/MS: *m/z* 379 [M + H]^+^.

### *N* ^*1*^,*N* ^*4*^-Bis(4-((5,6-Dimethyl-1*H*-Benzo[*d*][1,2,3]Triazol-1-yl)methyl)phenyl)Succinamide (8a)

Compound **8a** was obtained in 20% total yield; m.p. 270–272°C; TLC (chloroform/methanol 9.8/0.2); R_*f*_ = 0.31; ^1^H-NMR (200 MHz, DMSO-*d*_6_) *δ*: 10.02 (2H, s, 2NH), 7.78 (2H, s, 2H-4), 7.56 (2H, s, 2H-7), 7.54 (4H, d, *J* = 10.0, 2H-2′,6′), 7.23 (4H, d, *J* = 10.0, 2H-3′,5′), 5.82 (4H, s, 2CH_2_), 2.40 (4H, s, 2CH_2_), 2.35 (12H, s, 4CH_2_). ^13^C-NMR (DMSO-*d*_6_) *δ*: 170.3 (2CO), 144.6 (2C), 139.0 (2C), 137.3 (2C), 133.4 (2C), 131.7 (2C), 130.3 (2C), 128.2 (4CH), 119.1 (4CH), 118.2 (2CH), 109.6 (2CH), 50.5 (2CH_2_), 29.0 (CH_2_), 28.7 (CH_2_), 20.3 (2CH_3_), 19.8 (2CH_3_). C_34_H_34_N_8_O_2_; MW 586.69; Elem. Anal.: Calcd C 69.61, H 5.84, N 19.10 Found C 69.64, H 5.74, N 19.00. LC/MS: *m/z* 587 [M + H]^+^.

### 4-((4-((5,6-Dimethyl-1*H*-Benzo[*d*][1,2,3]Triazol-1-yl)methyl)phenyl)amino)-4-Oxobutanoic Acid (11a)

Compound **11a** was obtained in 10% total yield; m.p. 218–220 °C; TLC (chloroform/methanol 9/1); R_*f*_ = 0.41; ^1^H-NMR (200 MHz, DMSO-*d*_6_) *δ*: 10.02 (1H, s, NH), 7.78 (1H, s, H-4), 7.55 (1H, s, H-7), 7.53 (2H, d, *J* = 8.6, H-2′,6′), 7.23 (2H, d, *J* = 8.6, H-3′,5′), 5.80 (2H, s, CH_2_), 2.50 (4H, s, 2CH_2_), 2.34 (6H, s, CH_3_). ^13^C-NMR (DMSO-*d*_6_) *δ*: 174.1 (CO), 170.3 (CO), 144.6 (C), 139.1 (C), 137.3 (C), 133.4 (C), 131.6 (C), 130.2 (C), 128.1 (2CH), 119.0 (CH), 109.7 (CH), 50.5 (CH_2_), 32.0 (CH_2_), 31.3 (CH_2_), 19.8 (CH_3_), 18.6 (CH_3_). C_19_H_20_N_4_O_3_; MW 352.39; Elem. Anal.: Calcd C 64.76, H 5.72, N 15.90 Found C 64.72, H 5.72, N 15.80. LC/MS: *m/z* 353 [M + H]^+^.

### *N* ^*1*^,*N* ^*5*^ *-*Bis(4-((5,6-Dimethyl-1*H*-Benzo[*d*][1,2,3]Triazol-1-yl)methyl)phenyl)Glutaramide (8b)

Compound **8b** was obtained in 18% total yield; m. p. 253–255°C; TLC (chloroform/methanol 9.8/0.2); R_*f*_ = 0.54; ^1^H-NMR (200 MHz, DMSO-*d*_6_) *δ*: 9.95 (2H, s, 2NH), 7.79 (2H, s, 2H-4), 7.65 (2H, s 2H-7), 7.60 (4H, d, *J* = 8.6, H-2′,6′), 7.28 (4H, d, *J* = 8.6, 2H-3′,5′), 5.81 (4H, s, 2CH_2_), 2.42 (4H, t, 2CH_2_CO), 2.35 (12H, s, 4CH_3_), 1.86 (2H, m, CH_2_). ^13^C-NMR (DMSO-*d*_6_) *δ*: 171.7 (2CO), 144.0 (2C), 139.6 (2C), 137.8 (2C), 133.9 (2C), 132.0 (2C), 130.7 (2C), 128.6 (4CH), 119.7 (2CH), 119.6 (2CH), 118.5 (2CH), 109.6 (2CH), 51.0 (2CH_2_), 36.3 (2CH_2_), 21.7 (CH_2_), 20.7 (2CH_3_), 20.3 (2CH_3_). C_35_H_36_N_8_O_2_; MW 600.71; Elem. Anal.: Calcd C 69.98, H 6.04, N 18.65 Found C 70.00, H 6.21, N 18.70. LC/MS: *m/z* 601 [M + H]^+^.

### 5-((4-((5,6-Dimethyl-1*H*-Benzo[*d*][1,2,3]Triazol-1-yl)methyl)phenyl)amino)-5-Oxopentanoic Acid (11b)

Compound **11b** was obtained in 25% total yield; m.p. 270–272°C; TLC (chloroform/methanol 9/1); R_*f*_ = 0.35; ^1^H-NMR (200 MHz, DMSO-*d*_6_) *δ*: 10.35 (1H, s, NH), 7.78 (1H, s, H-4), 7.55 (3H, d, *J* = 8.6, H-7 e H-2′,6′), 7.23 (2H, d, *J* = 8.4, H-3′,5′), 5.80 (2H, s, CH_2_), 2.34 (6H, s, 2CH_3_), 2.02 (4H, t, 2CH_2_CO), 1.72 (2H, m, CH_2_). ^13^C-NMR (DMSO-*d*_6_) *δ*: 174.5 (CO), 170.9 (CO), 145.3 (C), 139.1 (C), 136.8 (2C) 132.5 (C), 130.1 (C), 128.6 (2CH), 124.3 (2CH), 122.0 (2CH), 50.7 (CH_2_), 35.4 (2CH_2_), 20.5 (CH_2_). C_20_H_22_N_4_O_3_; MW 366.41; Elem. Anal.: Calcd C 65.56, H 6.05, N 15.29 Found C 70.00, H 6.21, N 18.70. LC/MS: *m/z* 367 [M + H]^+^.

### *N* ^*1*^,*N* ^*6*^-Bis(4-((5,6-Dimethyl-1*H*-Benzo[*d*][1,2,3]Triazol-1-yl)methyl)phenyl)Adipamide (8c)

Compound **8c** was obtained in 23% total yield; m.p. 283–285°C; TLC (chloroform/methanol 9.8/0.2); R_*f*_ = 0.30; ^1^H-NMR (200 MHz, DMSO-*d*_6_) *δ*: 9.90 (2H, s, 2NH), 7.78 (2H, s, 2H-4), 7.57 (2H, s 2H-7), 7.52 (4H, d, *J* = 8.6, H-2′,6′), 7.23 (4H, d, *J* = 8.6, 2H-3′,5′), 5.81 (4H, s, 2CH_2_), 2.34 (12H, s, 4CH_3_), 2.25 (4H, t, 2CH_2_CO), 1.56 (4H, t, 2CH_2_). ^13^C-NMR (DMSO-*d*_6_) *δ*: 171.1 (2CO), 144.6 (2C), 139.0 (2C), 137.3 (2C), 133.4 (2C), 131.6 (2C), 130.3 (2C), 128.1 (4CH), 119.2 (4CH), 118.1 (2CH), 109.7 (2CH), 50.5 (2CH_2_), 36.2 (2CH_2_), 28.6 (2CH_2_), 20.3 (2CH_3_), 19.8 (2CH_3_). C_36_H_38_N_8_O_2_; MW 614.74; Elem. Anal.: Calcd C 70.34, H 6.23, N 18.23 Found C 70.14, H 6.28, N 18.23. LC/MS: *m/z* 615 [M + H]^+^.

### 6-((4-((5,6-Dimethyl-1*H*-Benzo[*d*][1,2,3]Triazol-1-yl)methyl)phenyl)amino)-6-Oxohexanoic Acid (11c)

Compound **11c** was obtained in 30% total yield; m.p. 203–204 °C; TLC (chloroform/methanol 9.5/0.5); R_*f*_ = 0.20; ^1^H-NMR (200 MHz, DMSO-*d*_6_) *δ*: 9.91 (1H, s, NH), 7.78 (1H, s, H-4), 7.69 (1H, s H-7), 7.55 (2H, d, *J* = 8.2, H-2′,6′), 7.23 (2H, d, *J* = 8.2, H-3′,5′), 5.81 (2H, s, CH_2_), 2.27 (6H, s, 2CH_3_), 2.24 (4H, m, 2CH_2_CO), 1.52 (4H, m, CH_2_CH_2_). ^13^C-NMR (DMSO-*d*_6_) *δ*: 174.8 (CO), 171.5 (CO), 145.0 (C), 139.5 (C), 137.8 (C), 133.9 (C), 132.1 (C), 130.8 (C), 128.6 (2CH), 119.7 (2CH), 118.6 (CH), 110.2 (CH), 50.9 (CH_2_), 36.5 (CH_2_), 33.8 (CH_2_), 25.0 (CH_2_), 24.6 (CH_2_), 20.8 (CH_3_), 20.3 (CH_3_). C_21_H_24_N_4_O_3_; MW 380.44; Elem. Anal.: Calcd C 66.30, H 6.36, N 14.73 Found C 66.30, H 6.46, N 15.00. LC/MS: *m/z* 381 [M + H]^+^.

### *N* ^*1*^,*N* ^*4*^-Bis(4-((5,6-Dimethyl-1*H*-Benzo[*d*][1,2,3]Triazol-1-yl)methyl)phenyl) Fumaramide (8d)

Compound **8d** was obtained in 10% total yield; m.p. 117–119°C; TLC (chloroform/methanol 9.8/0.2) R_*f*_ = 0.35; ^1^H-NMR (200 MHz, DMSO-*d*_6_) *δ*: 10.30 (1H, s, NH), 7.78 (2H, s, 2H-4), 7.68 (2H, s, 2H-7), 7.62 (4H, d, *J* = 8.6, 2H-2′,6′), 7.25 (4H, d, *J* = 8.6, 2H-3′,5′), 6.64 (2H, s, CH=CH), 5.80 (4H, s, 2CH_2_), 2.34 (12H, s, 4CH_3_). ^13^C-NMR (DMSO-*d*_6_) *δ*: 162.0 (2CO), 144.6 (2C), 138.4 (2C), 137.4 (2C), 134.0 (2CH), 133.5 (2C), 131.6 (2C), 131.5 (2C), 128.3 (4CH), 119.4 (4CH), 118.1 (2CH), 109.7 (2CH), 50.4 (2CH_2_), 20.4 (2CH_3_), 19.8 (2CH_3_). C_34_H_32_N_8_O_2_; MW 584.67; Elem. Anal.: Calcd C 69.85, H 5.52, N 19.17 Found C 69.85, H 5.52, N 19.17. LC/MS: *m/z* 585 [M + H]^+^.

### (*E*)-4-((4-((5,6-Dimethyl-1*H*-Benzo[*d*][1,2,3]Triazol-1-yl)methyl)phenyl)amino)-4-Oxobut-2-Enoic Acid (11d)

Compound **11d** was obtained in 20% total yield; m.p. 256–258°C; TLC (chloroform/methanol 8.5/1.5); R_*f*_ = 0.24; ^1^H-NMR (200 MHz, DMSO-*d*_6_) *δ*: 10.29 (1H, s, NH), 7.78 (1H, s, H-4), 7.65 (1H, s, H-7), 7.60 (2H, d, *J* = 8.6, H-2′,6′), 7.25 (2H, d, *J* = 8.6, H-3′,5′), 6.65 (2H, s, CH=CH), 5.82 (2H, s, CH_2_), 2.34 (6H, s, 2CH_3_). ^13^C-NMR (DMSO-*d*_6_) *δ*: 169.2 (CO), 164.1 (CO), 144.6 (C), 139.1 (C), 137.3 (C), 133.4 (CH), 131.6 (C), 130.6 (C), 128.3 (CH), 128.2 (CH), 119.4 (2CH), 118.1 (2CH), 109.7 (2CH), 50.5 (CH_2_), 20.3 (CH_3_), 19.8 (CH_3_). C_19_H_18_N_4_O_3_; MW 350,37; Elem. Anal.: Calcd C 65.13, H 5.18, N 15.99 Found C 65.18, H 5.25, N 15.78. LC/MS: *m/z* 351 [M + H]^+^.

### *N* ^*1*^,*N* ^*4*^-Bis(4-((5,6-Dimethyl-1*H*-Benzo[*d*][1,2,3]Triazol-1-yl)methyl)phenyl)Trephthalamide (8e)

Compound **8e** was obtained in 10% total yield; m.p. 250–252 °C; TLC (chloroform/methanol 9.8/0.2); R_*f*_ = 0.35; ^1^H-NMR (200 MHz, DMSO-*d*_6_) *δ*: 10.35 (2H, s, 2NH), 7.96 (2H, d, *J* = 7.8, H-2″, 6″) 7.79 (2H, s, 2H-4), 7.73 (4H, d, *J* = 8.2, 2H-2′,6′), 7.60 (2H, s, 2H-7), 7.57 (2H, d, *J* = 7.8, H-3″,5″), 7.30 (4H, d, *J* = 8.2, 2H-3′,5′), 5.86 (4H, s, 2CH_2_), 2.35 (12H, s, 4CH_3_). ^13^C-NMR (DMSO-*d*_6_) *δ*: 169.3 (2CO), 144.6 (2C), 139.4 (2C), 138.3 (2C), 136.5 (2C), 135.3 (2C), 131.6 (2C), 131.3 (C), 131.2 (C), 128.1 (4CH), 127.8 (2CH), 127.7 (2CH), 120.6 (4CH), 119.3 (2CH), 109.7 (2CH), 50.5 (2CH_2_), 19.8 (2CH_3_), 18.6 (2CH_3_). C_38_H_34_N_8_O_2_; MW 634.73; Elem. Anal.: Calcd C 71.91, H 5.40, N 17.65 Found C 71.75, H 5.62, N 17.56. LC/MS: *m/z* 635 [M + H]^+^.

### 4-((4-((5,6-Dimethyl-1*H*-Benzo[*d*][1,2,3]Triazol-1-yl)methyl)phenyl)carbamoyl)Benzoic Acid (11e)

Compound **11e** was obtained in 20% total yield; m.p. >300°C; TLC (chloroform/methanol 8.5/1.5); R_*f*_ = 0.37; ^1^H-NMR (200 MHz, DMSO-*d*_6_) *δ*: 10.43 (1H, s, NH), 8.00 (4H, s, H-2″,3″,5″,6″) 7.79 (1H, s, H-4), 7.74 (2H, d, *J* = 8.4, H-2′,6′), 7.61 (1H, s H-7), 7.31 (2H, d, *J* = 8.4, H-3′,5′), 5.86 (2H, s, CH_2_), 2.36 (6H, s, 2CH_3_). ^13^C-NMR (DMSO-*d*_6_) *δ*: 168.3 (CO), 165.6 (CO), 145.1 (C), 139.3 (C), 137.8 (C), 137.7 (C), 133.9 (2C), 132.1 (C), 131.7 (C), 129.6 (2CH), 128.5 (2CH), 128.0 (2CH), 121.1 (2CH), 118.6 (CH), 110.2 (CH), 51.0 (2CH_2_), 20.8 (CH_3_), 20.3 (CH_3_). C_23_H_20_N_4_O_3_; MW 400.43; Elem. Anal.: Calcd C 68.99, H 5.03, N 13.99 Found C 68.99, H 5.03, N 13.99. LC/MS: *m/z* 401 [M + H]^+^.

### *N* ^*2*^,*N* ^*6*^-Bis(4-((5,6-Dimethyl-1*H*-Benzo[*d*][1,2,3]Triazol-1-yl)methyl)phenyl)Pyridine-2,6-Dicarboxamide (8f)

Compound **8f** was obtained in 34% total yield; m.p. 186–188°C; TLC (chloroform/methanol 9.8/0.2); R_*f*_ = 0.50; ^1^H-NMR (200 MHz, DMSO-*d*_6_) *δ*: 11.08 (2H, s, 2NH), 8.46–8.43 (3H, m, H-3″,4″,5″), 7.93 (4H, d, *J* = 8.6, 2H-2′,6′), 7.88 (2H, s, 2H-4), 7.69 (2H, s 2H-7), 7.45 (4H, d, *J* = 8.6, 2H-3′,5′), 5.98 (4H, s, 2CH_2_), 2.44 (12H, s, 4CH_3_). ^13^C-NMR (DMSO-*d*_6_) *δ*: 162.0 (2CO), 148.6 (2C), 145.7 (2C), 144.9 (2C), 140.9 (2C), 138.9 (2C), 132.0 (2C), 131.6 (2C), 128.6 (2CH), 123.6 (4CH), 121.9 (4CH), 121.2 (1CH), 120.9 (2CH), 120.6 (2CH), 50.9 (2CH_2_). C_37_H_33_N_9_O_2_; MW 635.72; Elem. Anal.: Calcd C 69.90, H 5.23, N 19.83 Found C 69.98, H 5.36, N 19.83. LC/MS: *m/z* 636 [M + H]^+^.

### 6-((4-((5,6-Dimethyl-1*H*-Benzo[*d*][1,2,3]Triazol-1-yl)methyl)phenyl)carbamoyl)Picolinic Acid (11f)

Compound **11f** was obtained in 27% total yield; m.p. 214–216 °C; TLC (chloroform/methanol 8.5/1.5); R_*f*_ = 0.23; ^1^H-NMR (200 MHz, DMSO-*d*_6_) *δ*: 11.41 (1H, s, NH), 8.22–8.01 (3H, m, H-3″,4″,5″), 7.87 (2H, d, *J* = 8.6, H-2′,6′), 7.79 (1H, s, H-4), 7.62 (1H, s H-7), 7.32 (2H, d, *J* = 8.6, H-3′,5′), 5.87 (2H, s, CH_2_), 2.34 (6H, s, 2CH_3_). ^13^C-NMR (DMSO-*d*_6_) *δ*: 164.7 (CO), 161.6 (CO), 150.0 (2C), 148.2 (C), 144.3 (C), 140.1 (CH), 138.0 (C), 132.0 (C), 131.2 (C), 130.8 (C), 128.6 (CH), 128.5 (CH), 127.0 (CH), 125.8 (CH), 121.3 (CH), 120.9 (CH), 120.6 (CH), 112.5 (CH), 50.8 (CH_2_). C_22_H_19_N_5_O_3_; MW 401.42; Elem. Anal.: Calcd C 65.83, H 4.77, N 17.45 Found C 66.10, H 4.80, N 17.55. LC/MS: *m/z* 402 [M + H]^+^.

### *N* ^*2*^,*N* ^*5*^-Bis(4-((5,6-Dimethyl-1*H*-Benzo[*d]*[1,2,3]Triazol-1-yl)methyl)phenyl)Thiophene-2,5-Dicarboxamide (8g)

Compound **8g** was obtained in 10% total yield; m.p. 225–227°C; TLC (chloroform/methanol 9.8/0.2); R_*f*_ = 0.47; ^1^H-NMR (200 MHz, DMSO-*d*_6_) *δ*: 10.52 (2H, s, 2NH), 8.02 (2H, s, H-3″,4″), 7.79 (2H, s, 2H-4), 7.49 (4H, d, *J* = 7.8, 2H-2′,6′), 7.61 (2H, s, 2H-7), 7.31 (4H, d, *J* = 7.8, 2H-3′,5′), 5.87 (4H, s, 2CH_2_), 2.35 (12H, s, 4CH_3_). ^13^C-NMR (DMSO-*d*_6_) *δ*: 161.5 (2CO), 145.7 (2C), 144.6 (2C), 143.8 (2C), 137.9 (2C), 137.3 (2C), 136.3 (2C), 133.9 (2CH), 131.7 (2C), 131.5 (2C), 128.5 (2CH), 128.4 (2CH), 120.7 (2CH), 120.6 (2CH), 119.3 (2CH), 118.1 (2CH), 109.7 (2CH), 52.6 (2CH_2_), 20.4 (2CH_3_), 20.3 (2CH_3_). C_36_H_32_N_8_O_2_S; MW 640.76; Elem. Anal.: Calcd C 67.48, H 5.03, N 17.49 Found C 69.98, H 5.40, N 20.13. LC/MS: *m/z* 641 [M + H]^+^.

### 5-((4-((5,6-Dimethyl-1*H*-Benzo[*d*][1,2,3]Triazol-1-yl)methyl)phenyl)carbamoyl)thiophene-2-carboxylic Acid (11g)

Compound **11g** was obtained in 40% total yield; m.p. >300 °C; TLC (chloroform/methanol 8.5/1.5); R_*f*_ = 0.21; ^1^H-NMR (200 MHz, DMSO-*d*_6_) *δ*: 10.22 (1H, s, NH), 7.79 (2H, d, *J* = 3.8, H-3″,4″), 7.79 (1H, s, H-4), 7.68 (2H, d, *J* = 8.0, H-2′,6′), 7.60 (1H, s H-7), 7.29 (2H, d, *J* = 8.0, H-3′,5′), 5.86 (2H, s, CH_2_), 2.34 (6H, s, 2CH_3_). ^13^C-NMR (DMSO-*d*_6_) *δ*: 165.2 (CO), 160.2 (CO), 144.6 (C), 138.5 (C), 137.4 (2C), 133.5 (2C), 131.6 (C), 131.1 (C), 128.1 (2CH), 120.5 (2CH), 118.1 (2CH), 109.7 (2CH), 50.4 (CH_2_), 20.4 (CH_3_), 19.8 (CH_3_). C_21_H_18_N_4_O_3_S; MW 406.46; Elem. Anal.: Calcd C 62.05, H 4.46, N 13.78 Found 62.04, H 4.46, N 14.05. LC/MS: *m/z* 407 [M + H]^+^.

### *N* ^*1*^,*N* ^*4*^-Bis(4-((5,6-Dichloro-1*H*-Benzo[*d*][1,2,3]Triazol-1-yl)methyl)phenyl)Succinamide (9a)

Compound **9a** was obtained in 10% total yield; m.p. >300°C; TLC (chloroform/methanol 9.5/0.5); R_*f*_ = 0.70; ^1^H-NMR (400 MHz, DMSO-*d*_6_) *δ*: 10.03 (2H, s, NH), 8.48 (2H, s, H-4), 8.36 (2H, s, H-7), 7.55 (4H, d, *J* = 8.4, H-3′,5′), 7.31 (4H, d, *J* = 8.4, H-2′,6′), 5.90 (4H, s, CH_2_-N), 2.51 (4H, s, CH_2_-CH_2_). ^13^C-NMR (DMSO-*d*_6_) *δ*: 170.4 (2CO), 144.3 (2C), 139.2 (2C), 131.9 (2C), 130.7 (2C), 129.6 (2C), 128.5 (4CH), 127.2 (2C), 120.6 (2CH), 119.1 (2CH), 112.5 (4CH), 51.4 (2CH_2_-N), 29.0 (2CH_2_). C_30_H_22_Cl_4_N_8_O_2_; MW 668.36; Elem. Anal.: Calcd C 53.91, H 3.32, N 16.77 Found C 53.65, H 3.46, N 16.80 LC/MS: *m/z* 669 [M + H]^+^, 671 [M + H]^+^, 667 [M + H]^+^.

### 4-((4-((5,6-Dichloro-1*H*-Benzo[*d*][1,2,3]Triazol-1-yl)methyl)phenyl)amino)-4-Oxobutanoic Acid (12a)

Compound **12a** was obtained in 18% total yield; m.p. 214–216°C; TLC (chloroform/methanol 9.5/0.5); R_*f*_ = 0.38; ^1^H-NMR (400 MHz, DMSO-*d*_6_) *δ*: 10.29 (1H, s, NH), 8.48 (1H, s, H-4), 8.37 (1H, s, H-7), 7.55 (2H, d, *J* = 8.0, H-3′,5′), 7.31 (2H, d, *J* = 8.0, H-2′,6′), 5.90 (2H, s, CH_2_-N), 2.51 (4H, s, CH_2_CH_2_) ^13^C-NMR (DMSO-*d*_6_) *δ*: 176.9 (COOH), 171.6 (CONH), 144.2 (C), 139.5 (C), 131.8 (C), 130.7 (C), 129.3 (C), 128.5 (2CH), 127.2 (C), 120.5 (CH), 119.1 (2CH), 112.5 (CH), 50.9 (CH_2_-N), 33.0 (CH_2_), 32.0 (CH_2_). C_17_H_14_Cl_2_N_4_O_3_; MW 393.22; Elem. Anal.: Calcd C 51.93, H 3.59, N 14.25 Found C 51.73, H 3.60, N 14.25 LC/MS: *m/z* 417 [M + Na]^+^, 415 [M + Na]^+^, 393 [M + H]^+^.

### *N* ^*1*^,*N* ^*5*^-Bis(4-((5,6-Dichloro-1*H*-Benzo[*d*][1,2,3]Triazol-1-yl)methyl)phenyl)Glutaramide (9b)

Compound **9b** was obtained in 10% total yield; m.p. 204–206°C; TLC (chloroform/methanol 9.5/0.5); R_*f*_ = 0.70; ^1^H-NMR (400 MHz, DMSO-*d*_6_) *δ*: 9.94 (2H, s, NH), 8.48 (2H, s, H-4), 8.37 (2H, s, H-7), 7.56 (4H, d, *J* = 8.0, H-3′,5′), 7.31 (4H, d, *J* = 8.0, H-2′,6′), 5.90 (4H, s, CH_2_-N), 2.33 (4H, t, CH_2_CO), 1.87–1.85 (2H, m, -CH_2_-). ^13^C-NMR (DMSO-*d*_6_) *δ*: 170.8 (2CO), 144.3 (2C), 139.2 (2C), 131.9 (2C), 130.7 (2C), 129.7 (2C), 128.5 (4CH), 127.2 (2C), 120.6 (2CH), 119.3 (4CH), 112.5 (2CH), 50.9 (2CH_2_-N), 35.4 (2CH_2_), 20.8 (CH_2_). C_31_H_24_Cl_4_N_8_O_2_; MW 682.39; Elem. Anal.: Calcd C 54.56, H 3.54, N 16.42 Found C 54.46, H 3.60, N 16.26. LC/MS: *m/z* 685 [M + H]^+^, 681 [M + H]^+^.

### 5-((4-((5,6-Dichloro-1*H*-Benzo[*d*][1,2,3]Triazol-1-yl)methyl)phenyl)amino)-5-Oxopentanoic Acid (12b)

Compound **12b** was obtained in 15% total yield; m.p. 173–175°C; TLC (chloroform/methanol 9.5/0.05); R_*f*_ = 0.35; ^1^H-NMR (400 MHz, DMSO-*d*_6_) *δ*: 10.31 (1H, s, NH), 8.47 (1H, s, H-4), 8.36 (1H, s, H-7), 7.57 (2H, d, *J* = 8.0, H-3′,5′), 7.30 (2H, d, *J* = 8.0, H-2′,6’), 5.89 (2H, s, CH_2_-N), 2.29 (2H, t, CH_2_-CO-N), 2.05–2.01 (2H, m, CH_2_-COOH), 1.74 (2H, t, -CH_2_-). ^13^C-NMR (DMSO-*d*_6_) *δ*: 171.4 (2CO), 144.3 (C),139.4 (C), 131.9 (C), 130.7 (C), 129.5 (C), 128.5 (2CH), 127.2 (C), 120.5 (CH), 119.2 (2CH), 112.5 (CH), 50.9 (CH_2_-N), 36.0 (CH_2_), 35.5 (CH_2_), 21.5 (CH_2_). C_18_H_16_Cl_2_N_4_O_3_; MW 407.25; Elem. Anal.: Calcd C 53.09, H 3.96, N 13.76 Found C 52.86, H 4.16, N 14.14. LC/MS: *m/z* 447 [M + K]^+^, 445 [M + K]^+^.

### *N* ^*1*^,*N* ^*6*^-bis(4-((5,6-Dichloro-1*H*-Benzo[*d*][1,2,3]Triazol-1-yl)methyl)phenyl)Adipamide (9c)

Compound **9c** was obtained in 15% total yield; m.p. >300°C; TLC (chloroform/methanol 9.5/0.5); R_*f*_ = 0.63; ^1^H-NMR (400 MHz, DMSO-*d*_6_) *δ*: 9.92 (2H, s, NH), 8.48 (2H, s, H-4), 8.36 (2H, s, H-7), 7.55 (4H, d, *J* = 8.0, H-3′,5′), 7.31 (4H, d, *J* = 8.0, H-2′,6′), 5.90 (4H, s, CH_2_-N), 2.29 (4H, s, 2CH_2_CO), 1.58 (4H, s, CH_2_-CH_2_). ^13^C-NMR (DMSO-*d*_6_) *δ*: 171.1 (2C), 144.3 (2C), 139.3 (2C), 131.9 (2C), 130.7 (2C), 129.6 (2C), 128.5 (4CH), 127.2 (2C), 120.6 (2CH), 119.2 (4CH), 112.5 (2CH), 50.9 (2CH_2_-N), 36.2 (2CH_2_), 24.7 (2CH_2_). C_32_H_26_Cl_4_N_8_O_2_; MW 696.41; Elem. Anal.: Calcd C 55.19, H 3.76, N 16.09 Found C 55.10, H 3.80, N 15.89. LC/MS: *m/z* 697 [M + H]^+^, 695 [M + H]^+^.

### 6-((4-((5,6-Dichloro-1*H*-Benzo[*d*][1,2,3]Triazol-1-yl)methyl)phenyl)amino)-6-Oxohexanoic Acid (12c)

Compound **12c** was obtained in 13% total yield; m.p. 228–230°C; TLC (chloroform/methanol 9.5/0.5); R_*f*_ = 0.38; ^1^H-NMR (400 MHz, DMSO-*d*_6_) *δ*: 10.05 (1H, s, NH), 8.48 (1H, s, H-4), 8.37 (1H, s, H-7), 7.57 (2H, d, *J* = 8.0, H-3′,5′), 7.31 (2H, d, *J* = 8.0, H-2′,6′), 5.90 (2H, s, CH_2_-N), 2.27 (2H, t, CH_2_-CO), 2.12 (2H, t, CH_2_-CO), 1.58–1.49 (4H, m, CH_2_-CH_2_). ^13^C-NMR (DMSO-*d*_6_) *δ*: 171.3 (2CO), 144.3 (C), 139.3 (C), 131.9 (C), 130.7 (C), 129.6 (C), 128.5 (2CH), 127.2 (C), 120.6 (CH), 119.2 (2CH), 112.5 (CH), 50.9 (CH_2_-N), 36.1 (2CH_2_), 24.9 (CH_2_), 24.6 (CH_2_). C_19_H_18_Cl_2_N_4_O_3_; MW 421.28; Elem. Anal.: Calcd C 54.17, H 4.31, N 13.30 Found C 53.85, H 4.30, N 13.62. LC/MS: *m/z* 445 [M + Na]^+^, 443 [M + Na]^+^.

### *N* ^*1*^,*N* ^*4*^-Bis(4-((5,6-Dichloro-1*H*-Benzo[*d*][1,2,3]Triazol-1-yl)methyl)phenyl)Fumaramide (9d)

Compound **9d** was obtained in 10% total yield; m.p. >300°C; TLC (chloroform/methanol 9.5/0.5); R_*f*_ = 0.85; ^1^H-NMR (400 MHz, DMSO-*d*_6_) *δ*: 10.32 (2H, s, NH), 8.48 (2H, s, H-4), 8.38 (2H, s, H-7), 7.66 (4H, d, *J* = 8.0, H-3′,5′), 7.33 (4H, d, *J* = 8.0, H-2′,6′), 6.66 (2H, s, CH = CH), 5.92 (4H, s, CH_2_). ^13^C-NMR (DMSO-*d*_6_) *δ*: 164.4 (2CO), 144.3 (2C), 139.4 (2C), 131.9 (2C), 130.7 (2C), 129.9 (2C), 128.9 (6CH), 127.2 (2C), 120.8 (2CH), 119.5 (4CH), 112.7 (2CH), 50.9 (2CH_2_). C_30_H_20_Cl_4_N_8_O_2_; MW 666.34; Elem. Anal.: Calcd C 54.07, H 3.03, N 16.82 Found C 54.36, H 3.33, N 16.90. LC/MS: *m/z* 687 [M + Na]^+^, 667 [M + H]^+^, 665 [M + H]^+^.

### (*E*)-4-((4-((5,6-Dichloro-1*H*-Benzo[*d*][1,2,3]Triazol-1-yl)methyl)phenyl)mmino)-4-Oxobut-2-Enoic Acid (12d)

Compound **12d** was obtained in 10% total yield; m.p. 272–273°C; TLC (chloroform/methanol 9.5/0.5) R_*f*_ = 0.29; ^1^H-NMR (400 MHz, DMSO-*d*_6_) *δ*: 10.32 (1H, s, NH), 8.48 (1H, s, H-4), 8.38 (1H, s, H-7), 7.66 (2H, d, *J* = 8.4 Hz, H-3′,5′), 7.33 (2H, d, *J* = 8.4 Hz, H-2′,6′), 6.66 (2H, s, CH=CH), 5.92 (2H, s, CH_2_). ^13^C-NMR (DMSO-*d*_6_) *δ*: 164.5 (CONH), 163.9 (COOH), 145.2(C), 136.4 (HNCO-CH=), 134.6 (C), 134.2 (=CH-COOH), 133.5 (2C), 132.8 (C), 131.8 (C), 130.3 (2CH), 126.9 (2CH), 121.5 (2CH), 52.2 (CH_2_). C_17_H_12_Cl_2_N_4_O_3_; MW 391.21; Elem. Anal.: Calcd C 52.19, H 3.09, N 14.32 Found C 51.99, H 3.29, N 14.55. LC/MS: *m/z* 429 [M + K]^+^, 393 [M + H]^+^.

### *N* ^*1*^,*N* ^*4*^-Bs(4-((5,6-Dichloro-1*H*-Benzo[*d*][1,2,3]Triazol-1-yl)methyl)phenyl)Terephthalamide (9e)

Compound **9e** was obtained in 19% total yield; m.p. >300°C; TLC (chloroform/methanol 9/1); R_*f*_ = 0.54; ^1^H-NMR (400 MHz, DMSO-*d*_6_) *δ*: 10.43 (2H, s, NH), 8.48 (2H, s, 2H-4), 8.39 (2H, s, 2H-7), 8.05 (6H, s, H-3′,5′,2″,3″,5″,6″), 7.78 (2H, s, H-3′,5′), 7.39 (4H, s, 2H-2′,2H-6′), 5.95 (4H, s, 2CH_2_). ^13^C-NMR (DMSO-*d*_6_) *δ*: 166.7 (2CO), 164.9 (2C), 144.3 (2C), 138.9 (2C), 138.4 (2C), 131.9 (2C), 130.7 (2C), 130.7 (2C), 129.4 (2CH), 129.2 (4CH), 127.9 (4CH), 127.2 (2C), 120.6 (4CH), 112.5 (2CH), 50.9 (2CH_2_). C_34_H_22_Cl_4_N_8_O_2_; MW 716.40; Elem. Anal.: Calcd C 57.00, H 3.10, N 15.64 Found C 57.24, H 3.36, N 15.32. LC/MS: *m/z* 717 [M + K]^+^, 715 [M + H]^+^.

### *N* ^*2*^,*N* ^*6*^-Bis(4-((5,6-Dichloro-1*H*-Benzo[*d*][1,2,3]Triazol-1-yl)methyl)phenyl)Pyridine-2,6-Dicarboxamide (9f)

Compound **9f** was obtained in 13% total yield; m.p. >300°C; TLC (chloroform/methanol 9/1); R_*f*_ = 0.84; ^1^H-NMR (400 MHz, DMSO-*d*_6_) *δ*: 10.91 (2H, s, NH), 8.50 (2H, s, 2H-4), 8.43 (2H, s, 2H-7), 8.40–8.36 (1H, m, H-4″), 8.32–8.25 (2H, m, H-3″,5″), 7.82 (4H, d, *J* = 8.4, 2H-3′,5′), 7.45 (4H, d, *J* = 8.4, 2H-2′,6′), 5.99 (4H, s, 2CH_2_). ^13^C-NMR (DMSO-*d*_6_) *δ*: 162.0 (2CO), 148.6 (2C), 144.7 (2C), 143.4 (2C), 139.9 (2C), 138.9 (2C), 132.0 (2C), 131.6 (2C), 128.6 (2CH), 123.6 (4CH), 121.9 (4CH), 121.2 (1CH), 120.9 (2CH), 120.6 (2CH), 50.9 (2CH_2_). C_33_H_21_Cl_4_N_9_O_2_; MW 717.39; Elem. Anal.: Calcd C 55.25, H 2.95, N 17.57 Found C 55.00, H 3.10, N 17.78. LC/MS: *m/z* 756 [M + K]^+^, 718 [M + H]^+^.

### 6-((4-((5,6-Dichloro-1*H*-Benzo[*d*][1,2,3]Triazol-1-yl)methyl)phenyl)carbamoyl)Picolinic Acid (12f)

Compound **12f** was obtained in 40% total yield; m.p. 175–177°C; TLC (chloroform/methanol 9/1); R_*f*_ = 0.16; ^1^H-NMR (400 MHz, DMSO-*d*_6_) *δ*: 11.06 (1H, s, OH), 10.96 (1H, s, NH), 8.52 (1H, s, H-4), 8.45–8.41 (3H, t, *J* = 8.4, H-7, H-3′,5′), 8.36–8.31 (1H, m, H-4″), 7.94 (1H, d, *J* = 8.4, H-3″), 7.85 (1H, d, *J* = 8.4, H-5″), 7.50 (2H, d, *J* = 8.4, H-2′,6′), 6.03 (2H, s, CH_2_). ^13^C-NMR (DMSO-*d*_6_) *δ*: 164.7 (CO), 161.6 (CO), 149.0 (2C), 146.2 (C), 144.3 (C), 140.1 (CH), 138.0 (C), 132.0 (C), 131.2 (C), 130.8 (C), 128.6 (CH), 128.5 (CH), 127.0 (CH), 125.8 (CH), 121.3 (CH), 120.9 (CH), 120.6 (CH), 112.5 (CH), 50.8 (CH_2_). C_20_H_13_Cl_2_N_5_O_3_; MW 442.25; Elem. Anal.: Calcd C 54.32, H 2.96, N 15.84 Found C 54.32, H 3.06, N 15.74. LC/MS: *m/z* 443 [M + H]^+^, 441 [M + H]^+^.

### *N* ^*2*^,*N* ^*5*^-Bis(4-((5,6-Dichloro-1*H*-Benzo[*d*][1,2,3]Triazol-1-yl)methyl)phenyl)Thiophene-2,5-Dicarboxamide (9g)

Compound **9g** was obtained in 11% total yield; m.p. >300°C; TLC (chloroform/methanol 9/1); R_*f*_ = 0.82; ^1^H-NMR (400 MHz, DMSO-*d*_6_) *δ*: 10.43 (2H, s, NH), 8.49 (2H, s, 2H-4), 8.40 (2H, s, 2H-7), 8.01 (2H, s, H-3″,4″), 7.71 (4H, d, *J* = 8.4, 2H-3′,5′), 7.39 (4H, d, *J* = 8.4, 2H-2′,6′), 5.96 (4H, s, 2CH_2_). ^13^C-NMR (DMSO-*d*_6_) *δ*: 159.3 (2CO), 144.3 (2C), 143.9 (2C), 138.4 (2C), 134.1 (2C), 132.0 (2C), 130.9 (2C), 130.8 (2CH), 129.4 (4CH), 128.5 (2CH), 127.2 (2C), 120.7 (4CH), 112.5 (2CH), 50.8 (2CH_2_). C_32_H_20_Cl_4_N_8_O_2_S; MW 722.43; Elem. Anal.: Calcd C 53.20, H 2.79, N 15.51 Found C 52.88, H 3.00, N 15.67. LC/MS: *m/z* 761 [M + K]^+^, 721 [M + H]^+^.

## Results and Discussion

### Chemistry

**SCHEME 1:**
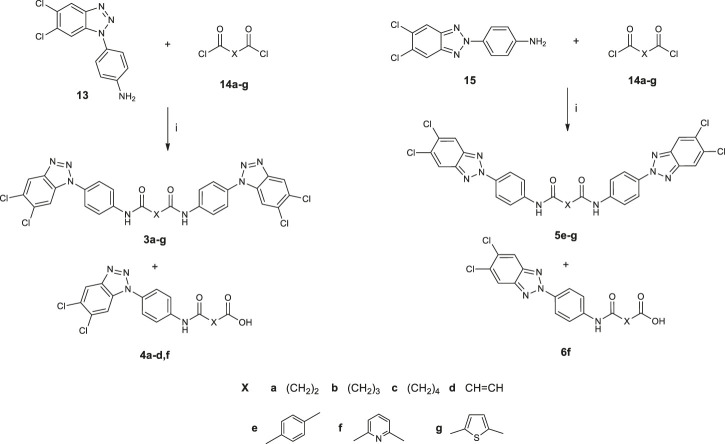
Synthesis of *N,N′*-bis [4-(1*H*(2*H*)-benzo [*d*][1,2,3]triazol-1(2)yl)phenyl]alkyl(aryl) dicarboxamides (**3a-g**; **5e-g**) and 4-((4-(5,6-dichloro-1*H*(2*H*)-benzo[*d*][1,2,3]triazol-1(2)-yl)phenyl)amino)-4-oxoalkyl(aryl)oic acids (**4a-d,f**; **6f**). 1) DMF, TEA, r.t., 3–72 h.

**SCHEME 2:**
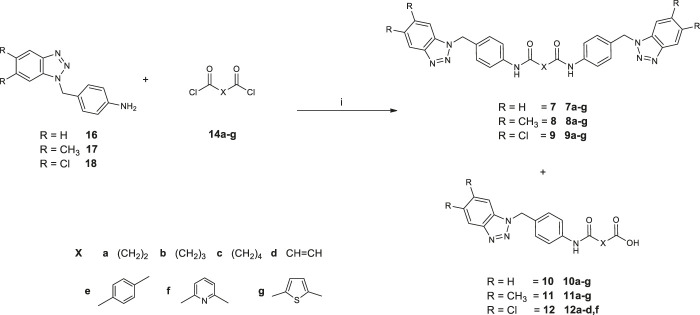
Synthesis of *N*,*N*′-bis[4-((5,6-*R*-1*H*-benzo[*d*][1,2,3]triazol-1-yl)methyl)phenyl]alkyl(aryl) dicarboxamides (**7a**-**g**; **8a**-**g**; **9a**-**g**) and 4-((4-((5,6-*R*-1*H*-benzo [*d*][1,2,3]triazol-1-yl)methyl)phenyl)amino)-4-oxoalkyl (aryl)oic acids (**10a**-**g**, **11a**-**g**; **12a**-**d**,**f**). 1) DMF, TEA, r.t., 3–72 h.

**SCHEME 3:**
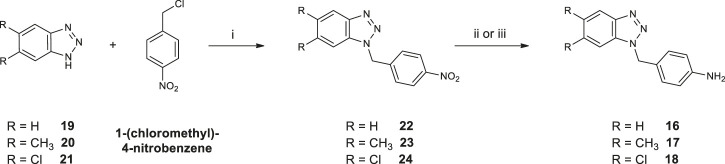
Synthesis of 4-((5,6-*R*-1*H*-benzo [*d*][1,2,3]triazol-1-yl)methyl)aniline (**16–18**). 1) Cs_2_CO_3_, DMF, 60°C for 48 h; 2) NH_2_NH_2_ (1:20), Pd/C (10% w/w), EtOH, 80°C for 2 h (to afford compounds **16** and **17**); 3) CH_3_NHNH_2_ (1:10), EtOH, 100°C for 48 h in autoclave (to afford compound **18**).

Synthetic pathways constructed to obtain the desired products are illustrated in Scheme 1 and Scheme 2, while Scheme 3 shows the synthetic route used to synthesize the benzyl-intermediates. 4- (5,6-dichloro-1(2)*H*-benzo [*d*][1,2,3]triazol-1-yl)aniline intermediates (**13** and **15**) were synthesized according to the previously described procedure (Carta et al., 2007). Diacyl-dichloride (**14a**-**g**) and 1-(chloromethyl)-4-nitrobenzene reagents were commercially purchased (Sigma-Aldrich). All the designed bis-benzotriazole-dicarboxamide derivatives were prepared by condensation of the corresponding benzotriazole-anilines (**13**,**15** and **16–18)** with the suitable diacyl dichloride (**14a-g**) in *N,N-*dimethylformamide (DMF) in the presence of triethylamine (TEA). These synthetic routes afforded the desired compounds bis-benzotriazole-dicarboxamides (**3a-g, 5e-g, 7a-g, 8a-g, 9a-g**) and the corresponding mono-substituted acidic derivatives (**4a-d**,**f**, **6f**, **10a-g**, **11a-g**, **12a-d,f**) as secondary products, that also were separated, purified and biologically evaluated. The bis-benzotriazole-dicarboxamides and the mono-substituted acidic derivatives were generally obtained in variable ratios. Intermediates **16**–**18** were obtained as depicted in Scheme 3, by a first substitution of the properly substituted benzotriazole (**19**–**21**) with 1-(chloromethyl)-4-nitrobenzene in basic conditions for triethylamine (TEA). The two isomers were separated from the 5,6-(*R*)-1 (2)-(4-nitrobenzyl)-1*H* (2*H*)-benzo [*d*][1,2,3]triazole mixture and the 5,6-(*R*)-1-(4-nitrobenzyl)-1*H*-benzo [*d*][1,2,3]triazoles (**22**–**24**) were subjected to reduction by two different synthetic pathways. Intermediates **16** and **17** were obtained by reduction of **22** and **23** respectively, with hydrazine and Palladium on carbon in ethanol, while compound **18** was gained by reduction of **24** solved in ethanol with methylhydrazine in the autoclave for 48 h.

### Antiviral activity

**FIGURE 3:**
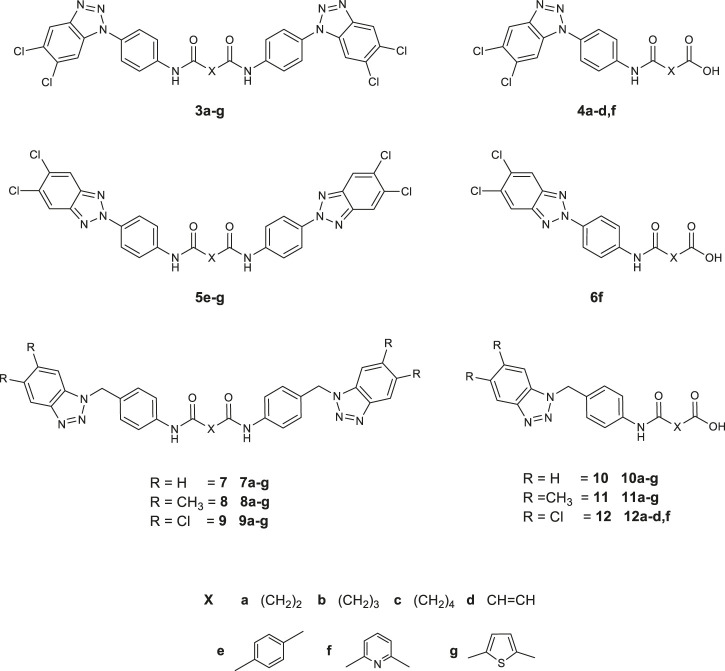
Synthesized derivatives, *N*,*N′*-bis [4-1*H* (2*H*)-benzo*[d]*[1,2,3]triazol-1 (2)-yl)phenyl] alkyl (aryl)dicarboxamides (series **3** and **5**) and *N*,*N′*-bis [4-1*H*-benzo*[d]*[1,2,3]triazol-1-yl) (methyl)phenyl]alkyl (aryl)dicarboxamides (series **7–9**). 4- (4-(5,6-dichloro-1*H*-benzo*[d]*[1,2,3]triazol-1 (2)-yl)phenyl)amino)-4-oxoalkyl (aryl)oic acids (series **4** and **6**), 4- (4-((5,6-R-1*H*-benzo*[d]*[1,2,3]triazol-1-yl)methyl)phenyl)amino)-4-oxoalkyl (aryl)oic acids (series **10–12**).

All the 56 synthesized compounds, depicted in Figure 3, were tested in a plaque-reduction assay for antiviral activity and in a cell-based assay for cytotoxicity. Viruses selected for the assay were Coxsackievirus B5 (CV-B5), a well-known human pathogen and the Poliovirus strain Sb-1, both belonging to the family of *Picornaviridae*. Compounds cytotoxicity was evaluated against three cell lines (Vero-76, MDBK, BHK-21). Table 1 shows the antiviral activity results and the corresponding cytotoxicity against the cell-line that supports viral replication, Vero-76. Results for the only active compounds are reported: derivatives **9a**, **9b** among the bis-benzotriazole-dicarboxamides and **4a**-**d** among the mono-substituted acidic derivatives. The remaining derivatives results are not reported in the table since they turned out not considerably active (EC_50_ values exceeded 100 µM). **9a** is the sole compound amidst the designed ones which turned out quite active against both the tested Picornaviruses (EC_50_ values of 23 and 43 µM, respectively). Surprisingly, the mono-substituted derivatives obtained as secondary products were found more active than the corresponding dicarboxamides. Compound **4c** was the most active against CV-B5, along with **4a** and **4d** with EC_50_ values ranging from 9 to 13 µM. The latter two are also endowed with poor or no cytotoxicity against Vero-76 cell line (CC_50_ 90 µM and >100 µM respectively).

**TABLE 1:** Antiviral activity and cytotoxicity results for compounds **9a**, **9b** among the bis-benzotriazole-dicarboxamides and **4a**-**d** among the mono-substituted acidic derivatives.

Dicarboxamides	*Cytotoxicity in Vero-76 cell-line*	*Efficacy in plaque reduction assay*		Mono-substituted	*Cytotoxicity in Vero-76 cell-line*	*Efficacy in plaque reduction assay*	
		CV-B5	Sb-1			CV-B5	Sb-1
	CC_50_ (µM)^a^	EC_50_ (µM)^b^			CC_50_ (µM)^a^	EC_50_ (µM)^b^	
9a	>100	23	43	4a	90	13	>90
9b	75	52	>75	4b	>100	45	44
				4c	37	9	>37
				4d	>100	10	>100
P	>100	0.005		P	>100	0.005	
2ec	>100		23	2ec	>100		23

Pleconaril (P) and 2′-C-ethynyl-cytidine (2ec) were used as control. Data represent mean values for three independent determinations. Variation among duplicate samples was less than 15%.

a Compound concentration (µM) required to reduce the viability of mock-infected Vero-76 cells by 50%, as determined by the MTT method.

b Compound concentration (µM) required to reduce the plaque number of CV-B5 or Sb-1 by 50% in Vero-76 monolayers.

### Antitumor activity

All synthesized compounds was also tested for antitumor activity against 7 cancer cell lines. For this purpose, human leukemia-lymphoma-derived cell lines CCRF-CEM, WIL-2NS, CCRF-SB and solid tumor-derived cell lines SK-MEL28, SK-MES 1, DU145, HeLa were selected. Fibroblast CRL 7065 cell line was used as control. Table 2 shows the results of the most promising compounds, derivatives **3b**, **3d** among bis-benzotriazole-dicarboxamides and **4d**, **9b** among mono-substituted acidic derivatives. In general terms, compound **3b** showed the widest range of activity reducing cell proliferation of six out of seven cell lines with CC_50_ ranging in the low micromolar values. Derivative **3d** had the lowest CC_50_ value against CCRF-CEM cell line (70 nM), while resulted inactive against most of the other tested cell lines. Among the mono-substituted acidic derivatives, **4d** was proved active when tested on human leukemia-lymphoma-derived cell lines while completely inactive against solid tumors proving an interesting selectivity of action. Compound **9b** is the one that turned out active against all the tested cancer cell lines, proving a wide range of action, showing, however, very low CC_50_ values. Dicarboxamides turned out slightly more cytotoxic when tested on CRL 7065 control cell line than the tested mono-substituted derivatives. To determine the cytotoxic selectivity of tested compounds, the selectivity index (SI) was calculated as a ratio of CC_50_ of non-tumor cells and CC_50_ of tumor cells. Since the non-specific mechanism of action of designed compounds SI values turned out very low, except for compound **3d**. The latter possessed CC_50_ values of 70 nM against tumor CCRF-CEM cell line and 370 nM against non-tumor CRL 7065 cell line, with a resulting SI of 5.3.

**TABLE 2:** Anti-proliferative activity for compounds **3b**, **3d**, **4d**, and **9b** against human leukemia-lymphoma-derived cell lines and solid tumor-derived cell-lines.

Compounds	CC_50_ (µM)^a^								
	*CCRF-CEM* ^b^	*WIL-2NS* ^c^	*CCRF-SB* ^d^	*SK-MEL28* ^e^	*SK-MES1* ^f^	*DU145* ^g^	*HeLa* ^h^	*CRL 7065* ^i^		
3b	0.5 ± 0.1	>100	0.35 ± 0.07	6.2 ± 1.3	6.8 ± 3.5	5.1 ± 0	7.1 ± 2.7	0.75 ± 0.05		
3d	0.07 ±0.02	>100	7.9 ± 0.9	>100	>100	>100	>100	0.37 ± 0.04		
4d	3.3 ±1.7	64 ± 10	0.51 ± 0.13	>100	>100	>100	>100	1.7 ± 0.1		
9b	5.5 ±1.8	23 ± 7	8.5 ± 3.2	2.6±0.7	9.9±3.1	5.2 ± 0.4	5.4 ± 0.7	6.7 ± 2.4		
Vincristine	0.003 ± 0.001	0.006 ± 0.002	0.003 ± 0.001	>4	0.01±0.02	0.02 ± 0.01	0.05 ± 0.02	>4		
Doxorubicin	0.02±0.01	0.06±0.01	0.03±0.02	1.1±0.2	0.65±0.05	0.3 ± 0.1	1.8 ± 0.2	2.0 ± 0.2		

Control is represented by CRL 7065 cell line. Vincristine and Doxorubicin were used as positive controls.

a Compound concentration required to reduce cell proliferation by 50%, as determined by the MTT method, under conditions allowing untreated controls to undergo at least three consecutive rounds of multiplication. Data represent mean values (±SD) for three independent determinations.

b CD4^+^ human acute T-lymphoblastic leukemia.

c Human splenic B-lymphoblastoid cells.

d Human acute B-lymphoblastic.

e Human skin melanoma.

f Human lung squamous carcinoma.

g Human prostate carcinoma.

h Human cervix carcinoma.

i Human foreskin fibroblasts.

**FIGURE 4:**
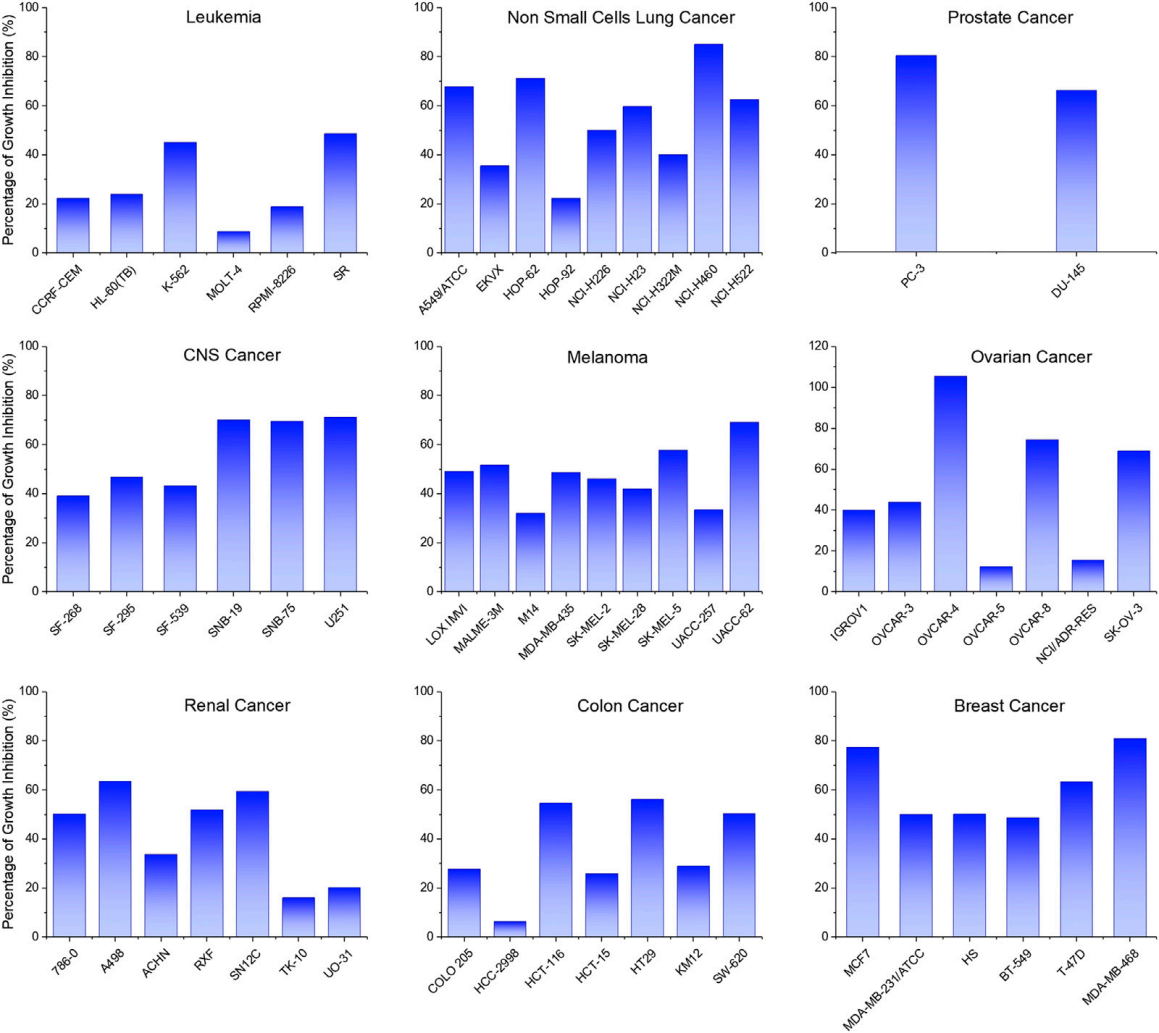
Antiproliferative screening assay: Percentage of Growth Inhibition (%) recorded on a panel of sixty cell lines treated with compound **3b** at 10 μM concentration.

The above-mentioned four promising compounds (**3b**, **3d**, **4d**, and **9b**) were then subjected to the NCI60 Human Tumor Cell Lines Screen (full tables of results in Supplementary Material). This screening was performed by the National *Cancer* Institute (NCI, Bethesda, United States), and evaluated our compounds in the anticancer assay at 10 μM concentration against a panel of 60 human tumor cell lines. The panel comprises a series of different cancer lines comprising hematological (leukemia) tumors and solid ones (non-small cell lung - NSCL, colon, central nervous system - CNS, renal, ovarian, breast and prostate cancers and melanoma). Among the tested compounds, **3b** turned out as the most active with the widest range of action. Results from the assays are reported as Percentage of Growth Inhibition (PGI) and are graphed in bar charts depicted in Figure 4. Cell lines are grouped per type of cancer. Our lead compound **3b** showed PGI values higher than 50% for 29 of the 60 cell lines, proving a wide antiproliferative activity. Best scores were recorded for breast cancer since most of the PGI values ranged from 48 up to 81%, and for prostate cancer (80 and 66% values of PGI against the two cell lines). Compound **3b** can be considered as an interesting antiproliferative agent against ovarian (OVCAR-8, SK-OV-3), CNS (SF-295, SNB19, SNB75, U251) and NSCL (A549/ATCC, HOP-62, NCI-H226, NCI-H23, NCI-H460, NCI-H522) cancers, while the growth of leukemia, melanoma, renal and colon cancer cells was less affected by the administration of derivative **3b**. The latter was also proved cytotoxic more than antiproliferative, against one ovarian cancer cell line (OVCAR-4) showing a PGI of 105.56%.

### Apoptosis Assay

**FIGURE 5:**
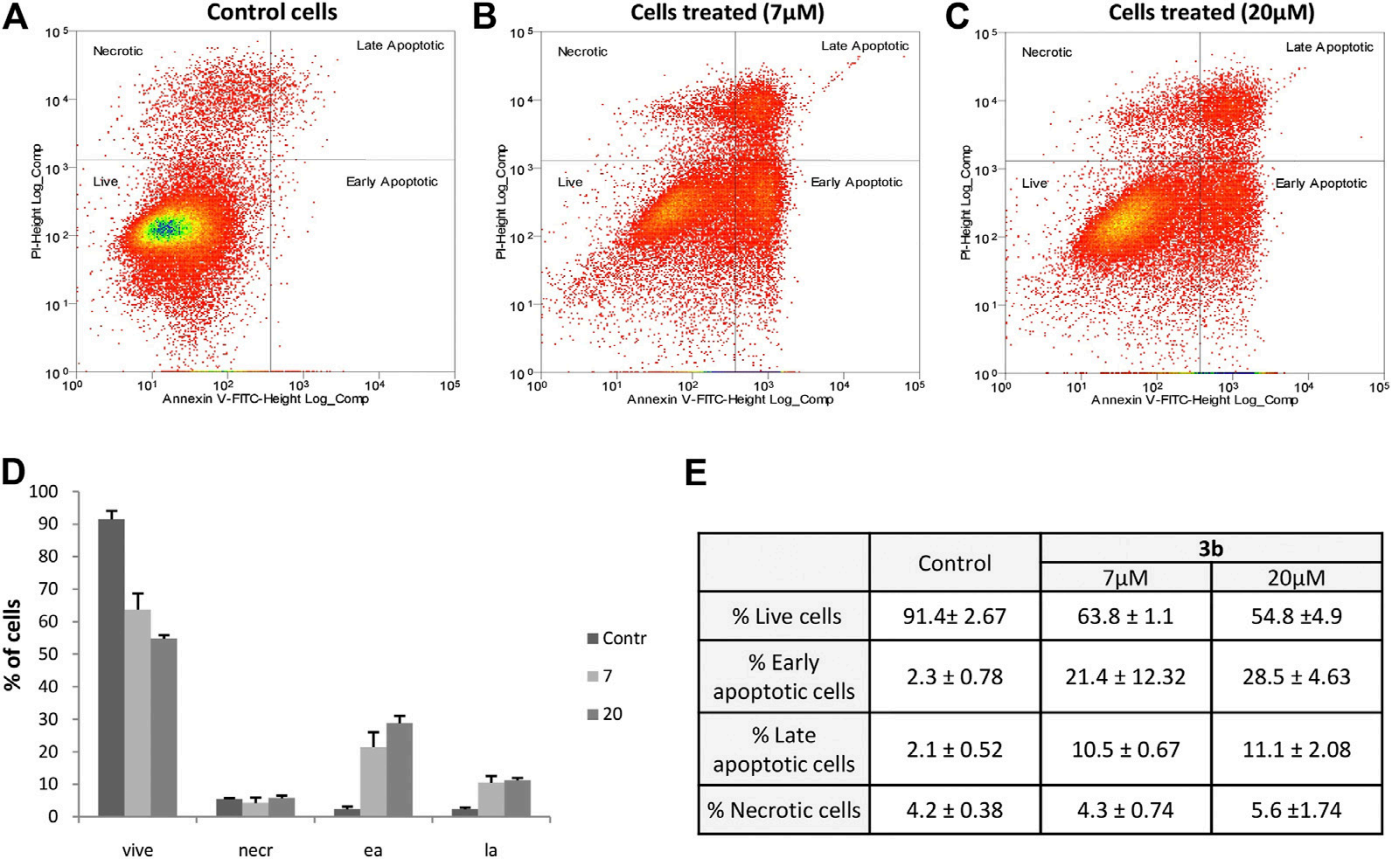
*N^1^*,*N^5^*-Bis(4-(5,6-dichloro-1*H*-benzo [*d*][1,2,3]triazol-1-yl)phenyl)glutaramide (**3b**) induced apoptosis in human lung carcinoma SK-MES 1 cells. The percentage of live, apoptotic, and necrotic cells were measured by flow cytometry using the PI-annexin V assay. Dot plots show cell death in SK-MES1 cells: control **(A)**, treated cells with 20 µM **(B)**, treated cells with 7 µM **(C)**. Percentage of live, apoptotic, and necrotic cells **(D,E)**. Each value represents the mean ± SD of independent experiments (*n* = 3).

Aiming to investigate the mechanism wherewith the cells die after lead compound administration, an apoptosis assay was performed. Data plots were produced by employing normalized fluorescent expression of Annexin V and PI (Figures 5A–C). Based on intensities distribution the percentage of living, early apoptotic, late apoptotic, and necrotic cells in untreated and treated conditions were analyzed. In the untreated cells (control) the majority (91.4%) of the cells were viable, the remaining cells died in the apoptotic and necrotic way (4.4 and 4.2%, respectively) (Figure 5A). Compound **3b** induced apoptosis after 96 h in a dose-dependent manner with 40% of apoptotic cells in 20 µm treated and 32% in 7 µm treated cells (Figures 5B,C, respectively). Particularly, Figures 5D,E clearly showed that most dead cells are characterized by early apoptotic features.

## Conclusion

We have designed and synthesized 56 new compounds, 31 bis-benzotriazole dicarboxamides (**3a-g**, **5e-g**, **7a-g**, **8a-g**, **9a-g**) and 25 corresponding mono-substituted acidic compounds (**4a-d**,**f**, **6f**, **10a-g**, **11a-g**, **12a-d**,**f**) as potential antiviral and/or antitumor agents, acting as false substrates. They were purified, characterized and tested for antiviral and antitumor activity in properly selected assays. Only a few of the synthesized compounds turned out as active against Coxsackievirus B5. Compounds **4a**, **4c,** and **4d** showed EC_50_ values ranging from 9 to 13 µM against CV-B5, while derivative **9a** is the sole compound among the bis-benzotriazole dicarboxamides that turned out quite active against both the tested Picornaviruses with EC_50_ values of 23 and 43 µM, against CV-B5 and Sb-1, respectively. From a selection of representatives that were subjected to an antitumor *in vitro* assay, four of them (**3b**, **3d**, **4d** and **9b**) resulted as the most interesting for their CC_50_ values that mostly turned out to be in the micromolar range. The same derivatives were tested by the National *Cancer* Institute on a panel of 60 human tumor cell lines. All the newly synthesized compounds that showed antiviral or anticancer activity bear two chlorine atoms on the BT scaffold (series **3**, **4** and **9**). Concerning the linker, the medium-length (-(CH_2_)_3_-) and the unsaturated (-CH=CH-) ones showed the best antitumor results (**3b**, **3d**, **4d**, and **9b**), while the aromatic linkers entail a complete loss of activity. 1*H*-BT moiety turned out to be the scaffold endowed with both biological activities (series **3**, **4**, and **9**). For the wide range of activity and the potency proved by the two screening assays, compound **3b** was selected as lead compound. When derivative **3b** was evaluated in apoptosis assay, results showed that the compound induced cell death by apoptosis in human lung tumor SK-MES 1. These findings will grant further studies involving this promising antiproliferative candidate. The latter will be used as a starting point for the next generation of compounds that will be the result of a proper structure modification process in order to increase the antiproliferative activity and potency. Alongside, compound **4c** will be used as a base to design new mono-substituted acidic compounds endowed with selective anti-Coxsackievirus activity.

## Data Availability

The original contributions presented in the study are included in the article/Supplementary Material, further inquiries can be directed to the corresponding authors.
