# Supplementary material for: Synthesis, Antitumor and Antiviral In Vitro Activities of New Benzotriazole-Dicarboxamide Derivatives
Source: Front Chem. 2021 May 4;9:660424. doi: 10.3389/fchem.2021.660424 (PMC8129498; doi:10.3389/fchem.2021.660424)
Supplement: Supplementary file 2 [file Presentation1.pdf]

Supplementary Material

1 Supplementary Table and Figures

Compound Label	*NSC Number*
**3b**	D-794940 / 1
**3d**	D-794941 / 1
**4d**	D-794939 / 1
**9b**	D-795499 / 1

Supplementary Table 1. Correlation of compound label and NSC number. The NSC numbers were assigned to each compound by the National Cancer Institute (NCI) before anticancer *in vitro* screening.

Developmental Therapeutics Program One Dose Mean Graph	NSC: D-794940 / 1	Conc: 1.00E-5 Molar	Test Date: Jan 03, 2017
	Experiment ID: 1701OS37		Report Date: Feb 14, 2018

Panel/Cell Line	Growth Percent
Leukemia	
CCRF-CEM	77.63
HL-60(TB)	75.93
K-562	54.85
MOLT-4	91.06
RPMI-8226	81.05
SR	51.24
Non-Small Cell Lung Cancer	
A549/ATCC	34.03
EKVX	64.42
HOP-62	28.04
HOP-92	77.59
NCI-H226	49.91
NCI-H23	40.22
NCI-H322M	59.89
NCI-H460	14.93
NCI-H522	37.36
Colon Cancer	
COLO 205	72.17
HCC-2998	93.71
HCT-116	45.29
HCT-15	74.11
HT29	43.76
KM12	70.92
SW-620	49.56
CNS Cancer	
SF-268	60.82
SF-295	53.13
SF-539	56.65
SNB-19	29.81
SNB-75	30.40
U251	28.65
Melanoma	
LOX IMVI	50.89
MALME-3M	48.24
M14	67.96
MDA-MB-435	51.21
SK-MEL-2	53.78
SK-MEL-28	57.92
SK-MEL-5	42.25
UACC-257	66.45
UACC-62	30.76
Ovarian Cancer	
IGROV1	59.80
OVCAR-3	56.08
OVCAR-4	-5.56
OVCAR-5	87.56
OVCAR-8	25.49
NCI/ADR-RES	84.49
SK-OV-3	31.00
Renal Cancer	
786-0	49.64
A498	36.48
ACHN	66.17
RXF 393	48.12
SN12C	40.50
TK-10	84.02
UO-31	79.77
Prostate Cancer	
PC-3	19.36
DU-145	33.58
Breast Cancer	
MCF7	22.55
MDA-MB-231/ATCC	49.87
HS 578T	49.76
BT-549	51.20
T-47D	36.68
MDA-MB-468	18.93
Mean	51.56
Delta	57.12
Range	99.27

Mean Growth Percent - Growth Percent

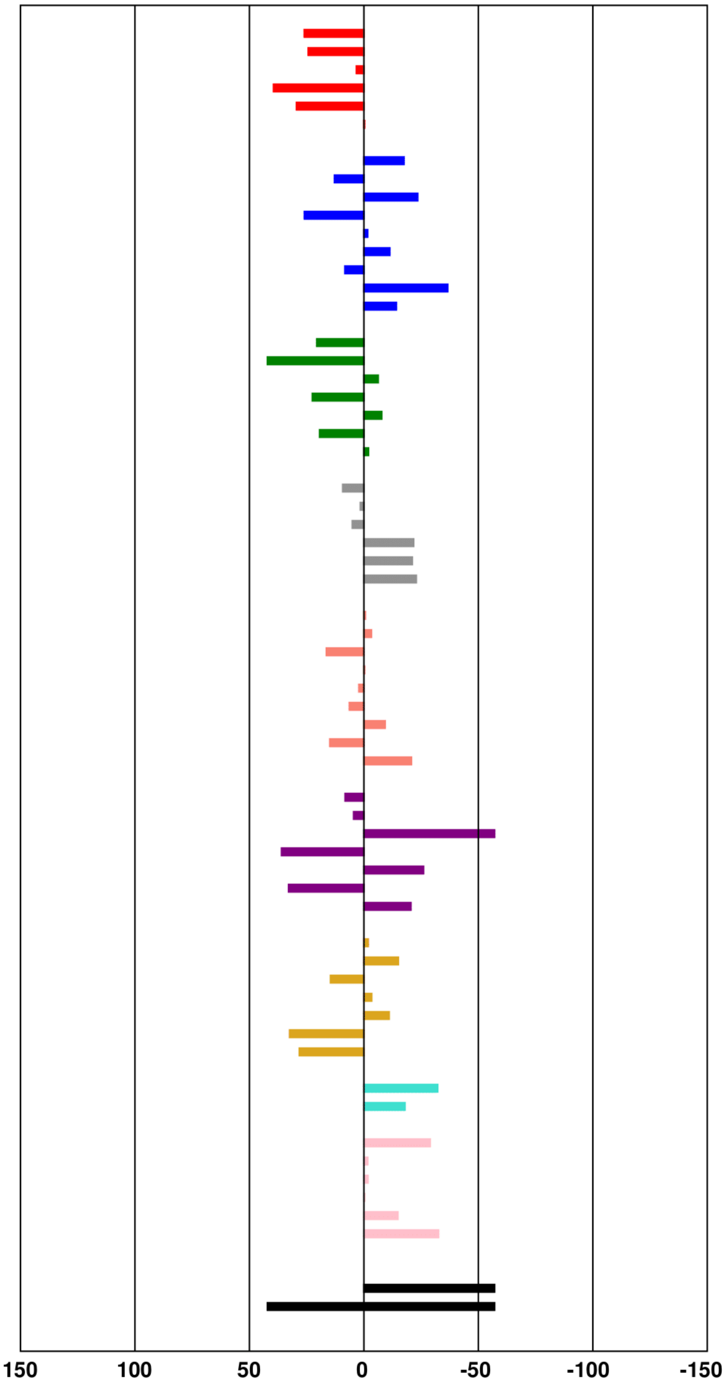

Supplementary Figure 2. NCI screening complete results for compound **3b**.

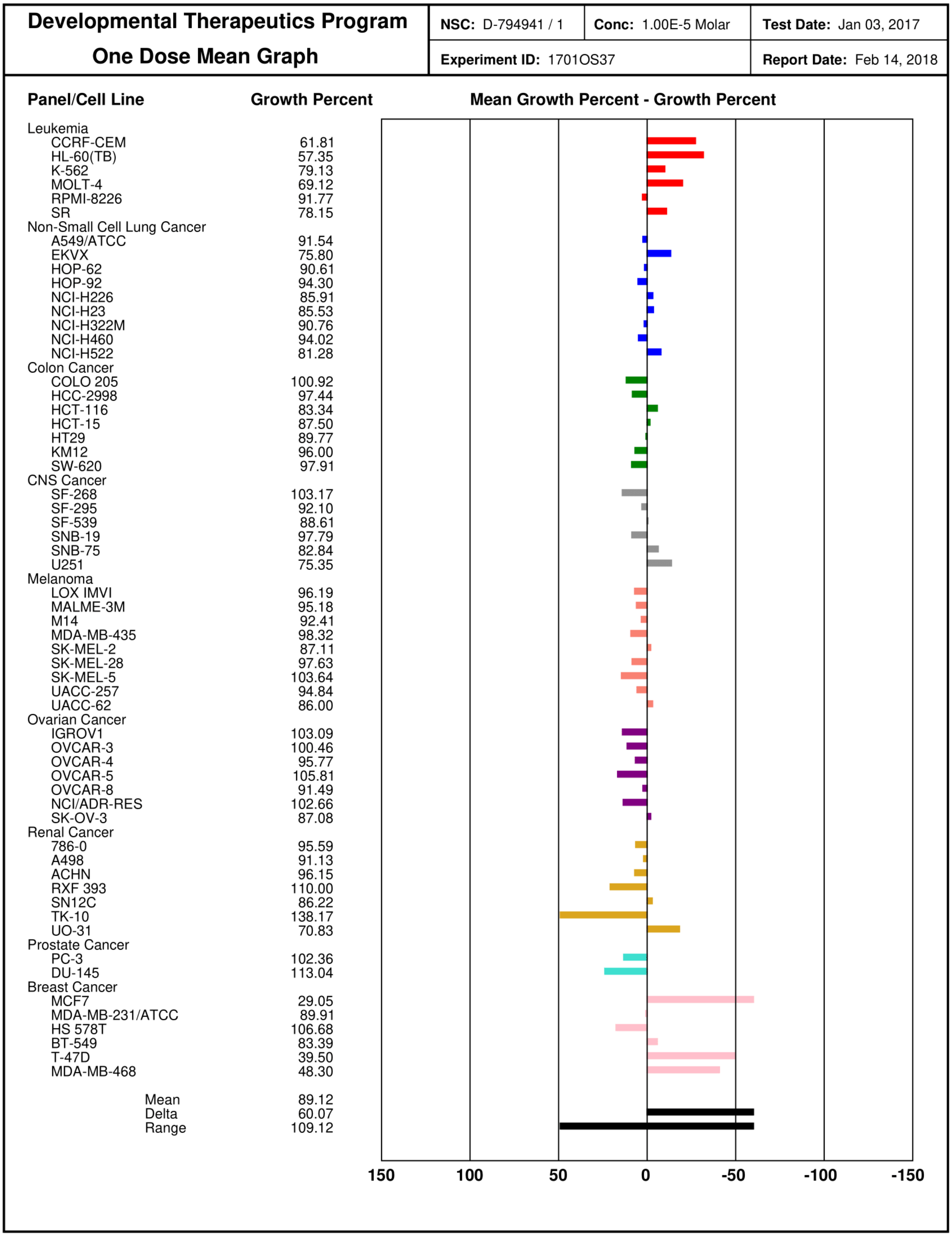

Supplementary Figure 2. NCI screening complete results for compound **3d**.

Developmental Therapeutics Program One Dose Mean Graph	NSC: D-794939 / 1	Conc: 1.00E-5 Molar	Test Date: Jan 03, 2017
	Experiment ID: 1701OS37		Report Date: Feb 14, 2018

Panel/Cell Line	Growth Percent
**Leukemia**	
CCRF-CEM	85.99
HL-60(TB)	74.63
K-562	84.16
MOLT-4	74.79
RPMI-8226	108.80
SR	76.68
**Non-Small Cell Lung Cancer**	
A549/ATCC	93.68
EKVX	80.85
HOP-62	101.13
HOP-92	97.61
NCI-H226	93.05
NCI-H23	92.23
NCI-H322M	90.48
NCI-H460	101.58
NCI-H522	80.85
**Colon Cancer**	
COLO 205	109.04
HCC-2998	101.66
HCT-116	91.76
HCT-15	97.75
HT29	100.95
KM12	89.35
SW-620	107.19
**CNS Cancer**	
SF-268	98.80
SF-295	94.78
SF-539	92.83
SNB-19	100.16
SNB-75	62.55
U251	92.76
**Melanoma**	
LOX IMVI	97.14
MALME-3M	99.92
M14	99.27
MDA-MB-435	94.89
SK-MEL-2	106.53
SK-MEL-28	101.26
SK-MEL-5	101.34
UACC-257	96.23
UACC-62	85.24
**Ovarian Cancer**	
IGROV1	97.32
OVCAR-3	93.23
OVCAR-4	88.07
OVCAR-5	105.13
OVCAR-8	95.48
NCI/ADR-RES	102.34
SK-OV-3	88.24
**Renal Cancer**	
786-0	99.34
A498	102.79
ACHN	101.20
RXF 393	123.52
SN12C	91.69
TK-10	149.82
UO-31	75.42
**Prostate Cancer**	
PC-3	96.93
DU-145	99.48
**Breast Cancer**	
MCF7	30.30
MDA-MB-231/ATCC	92.09
HS 578T	104.30
BT-549	77.03
T-47D	55.16
MDA-MB-468	62.83
Mean	93.04
Delta	62.74
Range	119.52

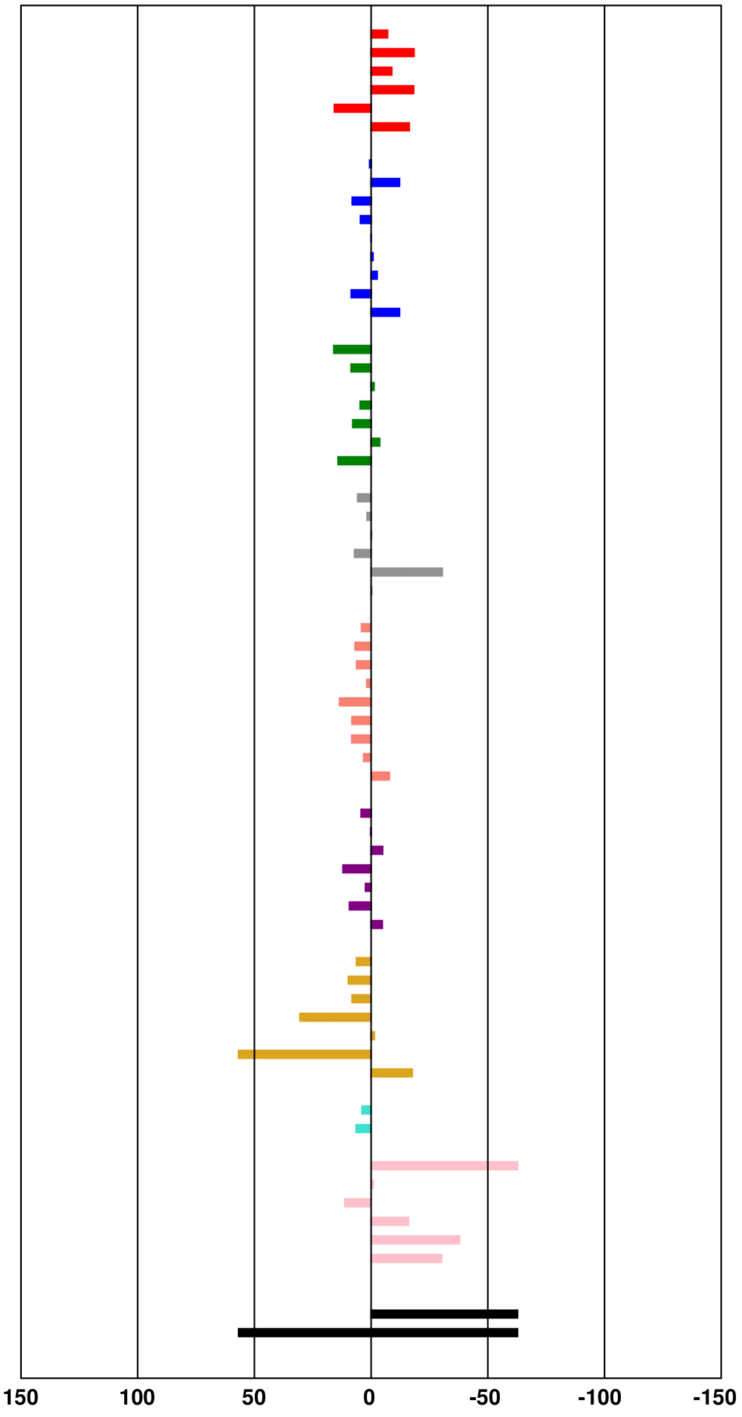

Supplementary Figure 3. NCI screening complete results for compound **4d**.

Developmental Therapeutics Program

One Dose Mean Graph

**NSC:** D-795499 / 1	**Conc:** 1.00E-5 Molar	**Test Date:** Jan 30, 2017
**Experiment ID:** 1701OS49		**Report Date:** Mar 23, 2017

Panel/Cell Line	Growth Percent
Leukemia	
CCRF-CEM	68.09
HL-60(TB)	89.72
K-562	66.97
MOLT-4	83.15
RPMI-8226	64.35
SR	79.17
Non-Small Cell Lung Cancer	
A549/ATCC	74.48
EKVX	75.62
HOP-92	64.68
NCI-H226	72.65
NCI-H23	62.17
NCI-H322M	74.02
NCI-H460	84.07
NCI-H522	62.43
Colon Cancer	
HCC-2998	90.19
HCT-116	75.10
HCT-15	97.27
HT29	86.46
KM12	79.92
SW-620	85.81
CNS Cancer	
SF-268	78.05
SF-295	71.91
SF-539	88.17
SNB-19	86.23
SNB-75	70.26
U251	84.20
Melanoma	
LOX IMVI	78.78
MALME-3M	97.03
M14	87.24
MDA-MB-435	87.84
SK-MEL-2	87.70
SK-MEL-28	102.52
SK-MEL-5	89.79
UACC-257	81.06
UACC-62	62.35
Ovarian Cancer	
IGROV1	81.11
OVCAR-3	87.74
OVCAR-4	65.57
OVCAR-5	95.80
OVCAR-8	76.27
NCI/ADR-RES	83.14
SK-OV-3	86.25
Renal Cancer	
786-0	85.33
A498	78.28
ACHN	83.82
RXF 393	93.69
SN12C	89.87
TK-10	106.80
UO-31	50.42
Prostate Cancer	
PC-3	60.18
DU-145	93.31
Breast Cancer	
MCF7	66.64
MDA-MB-231/ATCC	76.37
HS 578T	91.48
BT-549	71.47
T-47D	49.04
MDA-MB-468	59.06
Mean	79.32
Delta	30.28
Range	57.76

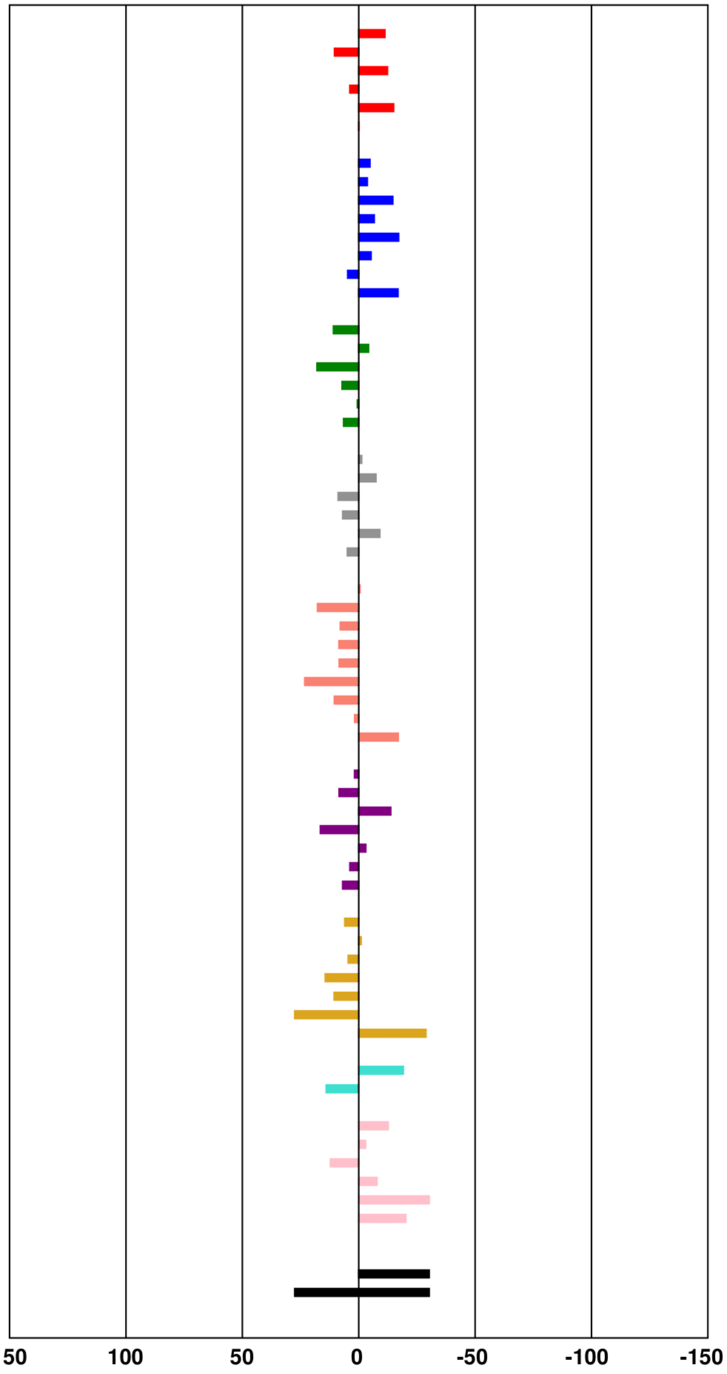

Supplementary Figure 4. NCI screening complete results for compound **9b**.
